# High‐permittivity Solvents Increase MXene Stability and Stacking Order Enabling Ultraefficient Terahertz Shielding

**DOI:** 10.1002/advs.202305099

**Published:** 2023-12-03

**Authors:** Xiaodan Hong, Zhenyu Xu, Zhong‐Peng Lv, Zhen Lin, Mohsen Ahmadi, Linfan Cui, Ville Liljeström, Volodymyr Dudko, Jiali Sheng, Xiaoqi Cui, Alexey P. Tsapenko, Josef Breu, Zhipei Sun, Qiang Zhang, Esko Kauppinen, Bo Peng, Olli Ikkala

**Affiliations:** ^1^ Department of Applied Physics Aalto University Espoo 02150 Finland; ^2^ Department of Electronics and Nanoengineering Aalto University Espoo 02150 Finland; ^3^ Nanomicroscopy Center OtaNano Aalto University Espoo 02150 Finland; ^4^ Bavarian Polymer Institute and Department of Chemistry University of Bayreuth D‐95447 Bayreuth Germany; ^5^ Honda Research Institute USA Inc. San Jose CA 95134 USA

**Keywords:** Janus film, MXene, permittivity, stability, terahertz shielding, X‐ray scattering

## Abstract

2D transition metal carbides and nitrides (MXenes) suggest an uncommonly broad combination of important functionalities amongst 2D materials. Nevertheless, MXene suffers from facile oxidation and colloidal instability upon conventional water‐based processing, thus limiting applicability. By experiments and theory, It is suggested that for stability and dispersibility, it is critical to select uncommonly high permittivity solvents such as *N*‐methylformamide (NMF) and formamide (FA) (*ε*_r_ = 171, 109), unlike the classical solvents characterized by high dipole moment and polarity index. They also allow high MXene stacking order within thin films on carbon nanotube (CNT) substrates, showing very high Terahertz (THz) shielding effectiveness (SE) of 40–60 dB at 0.3–1.6 THz in spite of the film thinness < 2 µm. The stacking order and mesoscopic porosity turn relevant for THz‐shielding as characterized by small‐angle X‐ray scattering (SAXS). The mechanistic understanding of stability and structural order allows guidance for generic MXene applications, in particular in telecommunication, and more generally processing of 2D materials.

## Graphical abstract

By using high permittivity solvents like *N*‐methylformamide (NMF) and formamide (FA), MXene stability and dispersibility are largely improved, enabling effective Terahertz (THz) shielding despite in ultrathin film. Small‐angle X‐ray scattering (SAXS) analysis highlights the importance of stacking order and mesoscopic porosity. This work offers valuable insights for generic MXene applications, particularly in telecommunication and 2D material processing.

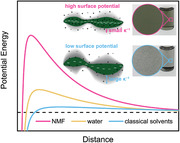

## 1 Introduction

2D transition metal carbides, nitrides, and carbonitrides, known as MXenes, have attracted major research interest, aiming at a wide range of applications, owing to their outstanding electronic, mechanical, catalytic, and optical properties.^[^ ^1^ ^]^ MXenes are usually synthesized from the MAX phase (M_n+1_AX_n_), using fluoride compounds by wet chemical or molten salt etching, forming 2D nanoflakes with formula M_n+1_X_n_T_x_ (*n* = 1–4),^[^ ^2^ ^]^ where M is the transition metal, A is an element from the group 13 or 14, X is carbon and/or nitrogen, and T_x_ represents oxygen (O, OH) and halogen (F, Cl, Br) surface termination groups, depending on the synthetic routes.^[^ ^3^ ^]^ Different from other inorganic 2D materials, e.g., graphene, transition metal dichalcogenides, and boron nitrides, MXenes with intrinsic hydrophilic surfaces have allowed processing in various polar solvents.^[^ ^4^ ^]^

Water is the most widely used solvent for dispersing delaminated MXene nanoflakes. However, water‐phase processing, even if potentially attractive for sustainability, severely limits practical exploitations due to oxidative instability and decreased conductivity, significantly shortening the lifetime of MXene.^[^ ^5^ ^]^ Another issue is the poor wettability of water on hydrophobic surfaces due to its high surface tension, which is unfavorable in forming heterostructures in certain applications, like ink printing.^[^ ^6^ ^]^ By contrast, etching or dispersing in an organic solvent has been proven a plausible way to prevent oxidation and enhance wettability.^[^ ^7^ ^]^ However, problems arise as pristine MXenes show severe aggregation in classical highly polar organic solvents such as alcohols, dimethyl sulfoxide, and dimethylformamide, which hinders extensive applications.^[^ ^7b^ ^]^ To resolve the stability and/or wetting issues, new strategies have been suggested, involving anti‐oxidation additives to MXenes aqueous dispersion, e.g., sodium *l*‐ascorbate^[^ ^8^ ^]^ or polyanionic salts for protecting the vulnerable edges of MXene nanoflakes.^[^ ^9^ ^]^ Surface coating using alkyl surfactants^[^ ^10^ ^]^ or quaternary ammonium cations^[^ ^11^ ^]^ can provide MXenes desired colloidal stability in organic solvents. However, antioxidants, intercalants, and surfactants deteriorate the intrinsic properties of MXenes.^[^ ^8^, ^12^ ^]^ Very recently, *N*‐methylformamide (NMF), has been suggested to solve the anti‐oxidation and anti‐humidity issues based on a high polarity index and H‐bonds with MXene terminations.^[^ ^13^ ^]^ Still, the generic mechanistic understanding of processing MXenes in solvents that can provide good colloidal stability, anti‐oxidation property, and wettability is not well explored, despite its importance for potential applications.

Recently, MXene has also been foreseen as feasible for future telecommunication applications.^[^ ^14^ ^]^ Owing to their metallic conductivity, flexibility, and processing ease in various media, MXenes could show great promise in the applications in microwave electromagnetic interference (EMI) shielding^[^ ^15^ ^]^ and in terahertz (THz) EMI for the next generation communication devices, taken that the stability issues could be solved.^[^ ^16^ ^]^ Due to the wavelength (0.03−3 mm) related to the THz electronics, properly structuring at the nano‐ or microscale is expected to be critical.^[^ ^17^ ^]^ To date, only a few works using Ti_3_C_2_T_x_‐based materials have shown improved THz shielding compared to traditional carbon‐based materials,^[^ ^18^ ^]^ using paints,^[^ ^19^ ^]^ sponges,^[^ ^20^ ^]^ and 3D foams.^[^ ^21^ ^]^ Grand challenges await therein to improve the shielding effectiveness (SE) and stability to translate to real applications.^[^ ^16^ ^]^ Also, new tools are needed to understand the assembled MXene micro–nano structures to further promote the THz SE.^[^ ^15^ ^]^

Herein, we demonstrate experimentally and theoretically that the solvent permittivity (*ε*) is the critical parameter 1) to solve the stability issue of MXene and 2) to allow well‐defined heterostructure formation upon combination with carbon nanotubes (CNT) for efficient THz shielding. NMF, (*ε* = 171 at 25 °C) and formamide (FA, *ε* = 109.5 at 25 °C) allow excellent dispersibility of Ti_3_C_2_T_x_, where deionized water (DIW, *ε* = 78.4 at 25 °C) and *N, N*´‐dimethylformamide (DMF, *ε* = 37.2 at 25 °C) are referenced as high polarity solvents with smaller permittivities. Here DMF is a control solvent being homologous to NMF and FA, as regarded as a good solvent for Ti_3_C_2_T_x_ among the classical polar organic solvents due to its relatively high permittivity and polarity index.^[^ ^7^, ^22^ ^]^ Importantly, casting from NMF allows improved stability and further flake stacking order in contrast to the reference solvents. As MXenes are vulnerable to water or humid environments,^[^ ^5^ ^]^ we will explore CNT combinations to construct MXene/CNT Janus films to reduce water sensitivity due to the water‐repellence of CNT. We explore THz electromagnetic shielding of the films and show that MXene/CNT Janus films using NMF processing are particularly effective. With our X‐ray scattering analysis method, the porosity originated from amorphous stacking to promote THz shielding is well‐elucidated.

## 2 Results and Discussion

### 2.1 High Permittivity Solvents for Both Effective Dispersants and Suppression of Ti_3_C_2_T_x_ Oxidation

**Figure 1:**
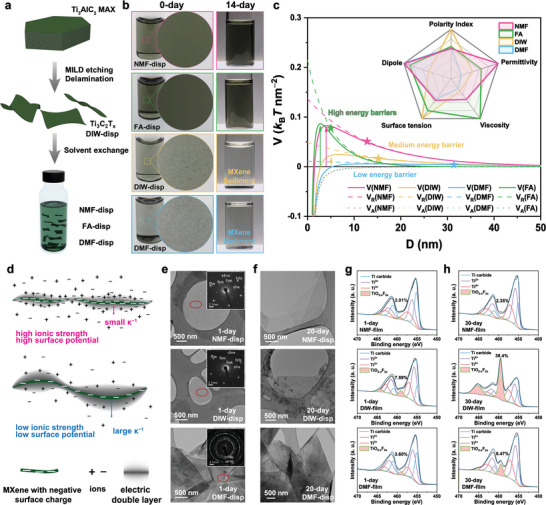
Schematic preparation of Ti_3_C_2_T_x_ dispersions and characterization of as‐prepared and aged Ti_3_C_2_T_x_ films upon casting from different solvents. a) Schematics of the MXene preparation and solvent exchange. b) Digital photo of diluted 0‐day (left) and 14‐day (right) NMF‐disp, FA‐disp, DIW‐disp, and DMF‐disp, at the concentrations of ≈0.016 mg mL^−1^. c) Schematic DLVO plots using planar charged surfaces model. The repulsion energy *V_R_* (color dash curves) and the attraction energy *V_A_* (color dot curves) are calculated from experimental data and solvent parameters (see Method S1, Supporting Information). The star symbols mark the Debye lengths κ^−1^ on *V_R_* curves. Inset: A radar chart for comparing different solvents. The values are normalized by the highest one in Table 1. d) Schematic illustration of MXene flakes in solvents with high (top) and low (bottom) permittivity. e, f) TEM images and selected area diffraction patterns (inset) of (e) 1‐day and (f) 20‐day Ti_3_C_2_T_x_ nanoflakes from NMF‐disp, DIW‐disp, and DMF‐disp. g, h) Ti 2p XPS spectra of (g) 1‐day and (h) 30‐day Ti_3_C_2_T_x_ MXene free‐standing films : NMF‐film, DIW‐film, and DMF‐film.

First, Ti_3_C_2_T_x_ MXene 2D nanoflakes were synthesized by selective etching of the Al‐layer of the Ti_3_AlC_2_ MAX phase, leading to their dispersion in DIW. The Ti_3_AlC_2_ aqueous dispersion is denoted as DIW‐disp, see **Figure** **1**a and Experimental Section. The X‐ray diffraction (XRD) pattern of the Ti_3_C_2_T_x_ casting from DIW‐disp (Figure S1a, Supporting Information) reveals successful etching and delamination, evidenced by the residual shifted (002) peak from 9.5^o^ to 6.9^o^, and the vanished peaks originated from Ti_3_AlC_2_ MAX phase.^[^ ^23^ ^]^ The atomic force microscopy (AFM) image (Figure S1b, Supporting Information) shows a typical single‐layered Ti_3_C_2_T_x_ flake on Si substrates with a diameter of ≈2 µm and a thickness of 3 nm.^[^ ^24^ ^]^ Scanning electron microscope (SEM) image of the few‐layered Ti_3_C_2_T_x_ on an anodic aluminum oxide (AAO) substrate (Figure S1c, Supporting Information) shows a similar size and morphology to the AFM image. More importantly, dispersions in NMF, FA, and DMF were subsequently achieved by solvent exchanges from water. Such dispersions are subsequently denoted as NMF‐disp, FA‐disp, and DMF‐disp, respectively. Diluted fresh samples of NMF and FA lead to visually clear dispersions without visible aggregation, whereas DIW and DMF show visible aggregation, surprisingly, despite their high solvent polarity index (Figure 1b). After standing for two weeks, no visible aggregation or precipitation in NMF‐disp and FA‐disp are observed, while DIW‐disp and DMF‐disp are fully precipitated on the bottom, qualitatively hinting towards better colloidal stability in NMF and FA. The dynamic light scattering (DLS) data (Figure S2, Supporting Information) suggest that the Ti_3_C_2_T_x_ nanoflakes have a larger lateral size (≈600 nm) in the dispersion of NMF and FA respectively than in DIW (500 nm) and DMF (250 nm). Further SEM images of Ti_3_C_2_T_x_ nanoflakes drop‐cast from the four solvents show a similar trend of the lateral sizes as from DLS, where NMF ≈ FA > DIW >> DMF (Figure S3, Supporting Information). The small lateral size in DMF again indirectly hints at lateral aggregation of the flakes.

In the state of the art, solvents with high polarity index, viscosity, permittivity, and surface tension, are believed to be critical to stably disperse MXenes.^[^ ^7b^ ^]^ NMF, FA, DIW, and DMF are selected as model solvents with their decreasing order of permittivity (**Table** **1**). Importantly, their other parameters, e.g., polarity indices, dipole moments, viscosities, and surface tensions, show different order. In the inset of Figure 1c, a radar chart to compare different solvent parameters among NMF, FA, DIW, and DMF underpins that even if all listed solvents are considered highly polar, their detailed properties are grossly different. In our system, only permittivity and viscosity follow the trend of colloidal stability. We then analyzed the colloid stability using the Derjaguin–Landau–Verwey–Overbeek (DLVO) theory (Method S1, Supporting Information) with measured data (Supporting Table S1). As calculated, the ionic strength of MXene dispersion in NMF and FA is much higher than in DIW and DMF, thus leading to a shorter Debye length (κ^−1^ = 12.9, 4.9, 15.1, and 31.4 nm for NMF, FA, DIW, and DMF, respectively) and a higher repulsion energy (*V_R_*) at a shorter distance between planar MXene flakes (color dash curves in Figure 1c). At the Debye length, the *V*_R_ has a trend of FA > NMF > DIW > DMF, consistent with zeta potential (ζ) measurement where ζ_FA_ = −37.8 mV, ζ_NMF_ = −36.3 mV, ζ_DIW_ = −29.0 mV and ζ_DMF_ = −25.9 mV (Figure S4, Supporting Information). The van der Waals attraction energies (*V*_A_) are related to the nature of the solvents and MXenes, however have very similar values for all solvents (color dot curves).^[^ ^35^ ^]^ The overall energy *V*  = *V*_A_  + *V_R_* is plotted (color solid curves), in which high energy barriers in NMF and FA are found, preventing MXene from self‐aggregation. This barrier becomes lower in DIW, which could be overcome with thermal movement. In DMF, there is no obvious barrier, explaining the severe aggregation proved by our experimental observation. We propose that solvents with high permittivity may provide higher energy barriers for MXene flakes and thus offer better colloidal stability. To verify this trend, other common organic solvents for dispersing MXenes with low permittivity, such as dimethylacetamide (DMAc, *ε* = 37.8 at 25 °C), dimethyl sulfoxide (DMSO, *ε* = 46.7 at 25 °C), and *N*‐methyl‐2‐pyrrolidone (NMP, *ε* = 32.7 at 25 °C) are also investigated (Method S1, Supporting Information). These solvents all show low energy barriers for MXene similar to DMF (Figure S5, Supporting Information), which reinforces our theory. Figure 1d summarizes the difference of the MXene colloid in solvents with high or low permittivity. The high permittivity NMF and FA have a better ability to screen and stabilize the surface charge of MXene flakes, leading to higher surface potentials and higher ionic strengths in the solution.^[^ ^36^ ^]^ We assume the highly charged Ti_3_C_2_T_x_ surface in NMF and FA led to more stretched and flat flakes, which explained its larger lateral size measured from DLS. The highly charged surface provides an energy barrier against aggregation and provides superior stability for MXene flakes. In addition, the high viscosity of NMF and FA could enhance the kinetic stability against colloidal aggregation and sediment.^[^ ^7b^ ^]^ Due to the excellent colloidal stability, we can achieve a Ti_3_C_2_T_x_ dispersion in NMF with a concentration of 40−60 mg mL^−1^, where the classical MXene organic solvent can only form MXene dispersion of ≈10 mg mL^−1^_._^[^ ^37^ ^]^

**Table 1:** Physical parameters of used solvents. Permittivity, polarity index, dipole moment, viscosity, and surface tension of NMF, FA, DIW, and DMF, as representatives for high, medium, and lower permittivity, are still all interpreted as highly polar solvents.

Properties	NMF	FA	DIW	DMF
Permittivity *ε*_r_ (298 K)	171^[^ ^25^ ^]^	109.5^[^ ^25^ ^]^	78.4^[^ ^26^ ^]^	37.2^[^ ^25^ ^]^
Polarity index^[^ ^27^ ^]^	6.0	9.6^a)^	10.2	6.4
Dipole moment *µ* (D)	3.86^[^ ^28^ ^]^	3.37^[^ ^28^ ^]^	1.87^[^ ^28^ ^]^	3.86^[^ ^29^ ^]^
Viscosity *η* (cp, 298 K)	1.65^[^ ^30^ ^]^	3.31^[^ ^31^ ^]^	0.89^[^ ^32^ ^]^	0.803^[^ ^32^ ^]^
Surface tension (mN m^−1^, 298 K)	38.0^[^ ^33^ ^]^ (303 K)	58.5^[^ ^33^ ^]^	72.0^[^ ^34^ ^]^	35.2^[^ ^33^ ^]^

^a)^ approximate value.

For further investigations, NMF, DIW, and DMF are selected as the representative solvents. Transmission electron microscopy (TEM) images in Figure 1e show drop‐cast samples of as‐prepared dispersions in NMF, DIW, and DMF, respectively. We found well‐dispersed few‐layered Ti_3_C_2_T_x_ nanoflakes with clear boundaries and flat surfaces in NMF and DIW. However, MXene cast from DMF shows clear aggregation into randomly stacked flakes. The insets in Figure 1e depict selected area diffraction patterns. Samples cast from NMF and DIW show clear diffraction spots indicating a well‐defined in‐plane crystallinity in Ti_3_C_2_T_x_ flakes. By contrast, the sample cast from DMF shows a diffraction ring, resulting from severe aggregation or folding of Ti_3_C_2_T_x_ flakes. After storing the DIW‐disp in the refrigerator under N_2_ atmosphere and NMF/DMF‐disp under ambient conditions for 20 days, Ti_3_C_2_T_x_ nanoflakes cast from NMF and DMF show no clear changes compared to the corresponding 1‐day samples (Figure 1f). However, nanoparticles were observed on the edges of Ti_3_C_2_T_x_ flakes in DIW‐based samples upon aging, suggesting instability even under N_2_ protection. From energy‐dispersive X‐ray spectroscopy and high‐resolution TEM image (Figure S6, Supporting Information), we conclude that these nanoparticles are likely TiO_2_ as a degradation product of Ti_3_C_2_T_x_.^[^ ^5^ ^]^

More quantitatively, we next employed X‐ray photoelectron spectroscopy (XPS) to study the composition and oxidation of the cast films during aging. Survey spectra and deconvolution results of F, O, and C are plotted in Figures S7 and S8, Supporting Information. In the Ti 2p region (Figure 1g), three doublets of asymmetric peaks related to the Ti_3_C_2_ structure centered at ≈455.0 eV (blue), 455.8 eV (purple), and 457.2 eV (red) were found. The peak centered at ≈459.2 eV was assigned to oxidized TiO_2−x_F_2x_,^[^ ^38^ ^]^ rendering quantitative analysis of the oxidization degree. For the 1‐day films, MXene film cast from NMF (denoted as NMF‐film) has a TiO_2−x_F_2x_ peak percentage of 2.01%, while for films cast from DIW (DIW‐film) and DMF (DMF‐film) are 7.59% and 3.60%, respectively, indicating the lowest oxidation degree of NMF‐film. After being stored in the ambiance for 30 days (Figure 1h), there is only a slight increase of TiO_2−x_F_2x_ (from 2.01% to 2.35%) in NMF‐film, whereas a considerable increase from 3.6% to 8.47% in DMF‐film was observed. However, the TiO_2−x_F_2x_ peak percentage increases dramatically from 7.59% to 38.4% in DIW‐film, indicating an easy surface oxidization. We suggest that the intercalated water which is difficult to remove by vacuum drying plays an important role in oxidization, as the oxidization of Ti_3_C_2_T_x_ is a synergistic effect of both water and oxygen.^[^ ^5^ ^]^ The robust antioxidation ability of NMF‐film originates from the strong hydrogen bonding between intercalated NMF and MXene termination groups.^[^ ^13^ ^]^ These results show that NMF is a superior dispersant versus water and DMF for preserving Ti_3_C_2_T_x_ MXene both in dispersion and dry films since it provides superior dispersibility and suppresses oxidation simultaneously.

### 2.2 Fabrication of MXene/CNT Janus Film

**Figure 2:**
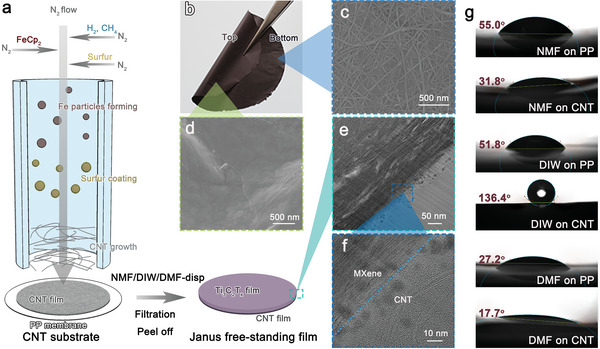
Schematic preparation of free‐standing MXene/CNT Janus film and morphological characterization. a) Fabrication of the MXene/CNT Janus free‐standing film. b) Photograph of the top and bottom surface of MXene/CNT Janus film. c, d) SEM images of MXene/CNT Janus film on (c) CNT face and (d) MXene face. e, f) TEM images of cross‐sectional MXene/CNT Janus film prepared by FIB. g) Contact angles of NMF, DIW, and DMF measured on PP membrane and CNT substrate.

We prepared asymmetric Janus films by solvent‐casting of MXene on CNT substrates, as we expected such a process to allow an intimate colloidal‐level heterostructural connection between them, see **Figure** **2**a and Experimental Section. At first, CNT film was deposited on a polypropylene (PP) membrane, using a floating catalyst chemical vapor deposition growth method.^[^ ^39^ ^]^ The CNT film is made of few‐wall carbon nanotubes with ≈3 nm in diameter, showing an interwoven geometry and 88% transmittance at 550 nm (Figure S9, Supporting Information).

MXene/CNT Janus film was then fabricated by a vacuum‐assisted filtration method. Typically, Ti_3_C_2_T_x_ MXene dispersions in NMF, DMF, or DIW, were filtrated on top of the CNT substrate on a PP membrane. After drying, the free‐standing MXene/CNT Janus film can be easily peeled off from the PP membrane denoted as NMF‐film/CNT, DIW‐film/CNT, and DMF‐film/CNT. Such films can optionally be processed subsequently for various coating and painting processes. As the photo of the highly flexible MXene/CNT Janus film shown in Figure 2b, the MXene face (top) has a glossy metallic color, while the CNT face (bottom) is black. In the SEM images of its top and bottom faces, the CNTs network (Figure 2c) and stacked MXene flakes (Figure 2d) were observed respectively, proving the asymmetric Janus structure. The cross‐section of the MXene layer cutted with a focus ion beam (FIB) (Figure 2e) shows a characteristic accordion structure with no Ti_3_C_2_T_x_ flakes in the CNT layer. Interestingly, we also observe some brighter spindle‐shaped “pores” of a few tens of nanometers of their long axis between MXene stacks. There is no obvious lattice periodicity in these pores, indicating an amorphous nature. From the high magnification cross‐sectional TEM image (Figure 2f), we can clearly find the boundary of MXene and CNT and the thickness of the CNT film is ≈50 nm. Different from the hydrophilic liquid phase produced CNT with short average length (≈1 µm) low conductivity and low mechanical strength, our CNTs are much longer (≈20 µm) with a hydrophobic surface and excellent mechanical properties (strength > 500 MPa) and conductivity (≈4000 S cm^−1^),^[^ ^40^ ^]^ which is favorable to fabricate high‐performance ultrathin Janus film. The CNT preparation is also scalable, leading to the possibility of large‐scale production of the Janus film. We further test the contact angles of NMF, DIW, and DMF on PP membranes and CNT film substrates (Figure 2g). All the solvents can nicely wet the hydrophilic PP surface. However, for hydrophobic CNT surface, only NMF and DMF with their lower surface tension have proper wettability. The result suggests that NMF is a promising dispersant for fabricating MXene heterostructures on various surfaces due to the combination of excellent dispersibility and wettability, which is missing in water or conventional polar organic solvents.

### 2.3 Structural Studies of Ti_3_C_2_T_x_ Stacked Janus Films with CNT

**Figure 3:**
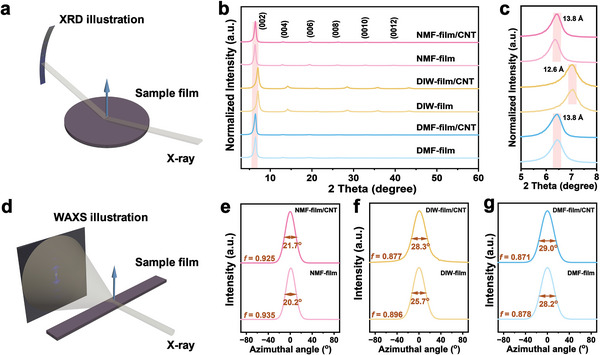
Structural studies of MXene and asymmetric MXene/CNT films using wide‐angle X‐ray methods. a) Schematic illustration of the XRD measurement setup for the free‐standing films. b, c) XRD patterns of (b) MXene and MXene/CNT films, and (c) enlarged diffraction patterns of the (002) peak of the films in the 2*θ* range from 5−8°. d) Schematic illustration of the WAXS measurement setup for the free‐standing films. e–g) Azimuthal plot for the (002) peaks in azimuth range of −90° and 90° for (e) NMF‐film and NMF‐film/CNT, (f) DIW‐film and DIW‐film/CNT, and (g) DMF‐film and DMF‐film/CNT. Both *f* and full width at half maximum values are given. The blue arrows in (a) and (d) indicate the *c*‐axis of the lamellar structure of MXene.

To understand the dominant features at different length scales, we characterize the structures of all three types of Janus films and the reference pure MXene films using X‐ray scattering and electron microscopy methods. **Figure** **3**a illustrates the XRD measurement setup in symmetric reflection geometry, where the lamellar axis *c* of the samples is normal to the plane of reflection. In Figure 3b, all films show well‐defined lamellar structures, indicated by the existence of high‐order lamellar peaks up to (0012). However, in DMF‐film and DMF‐film/CNT, the intensities of the high‐order peaks decrease faster than those in the NMF and DIW samples. This suggests a larger lattice displacement in samples cast from DMF.^[^ ^41^ ^]^ From the SEM image of the cross‐section cut by FIB (Figure S10, Supporting Information), films cast from DMF indeed show more disordered packing and porous defects compared to those from NMF and from DIW. From Figure 3c, the calculated *d*‐spacing from the (002) peak in samples cast from DMF and NMF is ≈13.8 Å, higher than that from DIW (≈12.6 Å), which is caused by the larger molecule size of the organic intercalant than water. Furthermore, the *d*‐spacing is independent of the presence of the CNT, consistent with our observation of phase separation in cross‐sectional TEM (Figure 2f).

Figure 3d shows the setup of the wide‐angle X‐ray scattering (WAXS) measurement used for evaluating structural anisotropy in terms of Herman's orientation factor (*f*) (Method S2, Supporting Information),^[^ ^42^ ^]^ where the X‐ray beam is aligned perpendicularly to the *c*‐axis. Here we use the (002) peaks from the 2D scattering patterns for the azimuthal profile analysis (see the detailed 2D WAXS patterns in Figure S11, Supporting Information). As shown in Figure 3e–g, the NMF‐film shows a much higher *f* value (0.935) than DIW‐film (0.896) and DMF‐film (0.878), indicating its best alignment of MXene flakes along the film plane. We assume that MXene colloidal stability (Figure 1c) and solvent wettability (Figure 2g) can jointly affect the alignment factor. NMF wets PP properly and provides the best colloidal stability among the studied solvents, leading to the highest *f* value. Although DMF shows the smallest wetting angle on PP, the *f* value of DMF‐film is low due to the poor dispersibility of Ti_3_C_2_T_x_. Not surprisingly, all *f* factors decrease when forming MXene/CNT Janus films, because of the distorted stacking of MXene flakes while deposited to the rough surface of the CNT network. However, the *f* values only decrease slightly for NMF‐film/CNT and DMF‐film/CNT due to good solvent wettability on CNT substrate, while there is a major decline for DIW‐film/CNT, caused by poor wettability of water.

**Figure 4:**
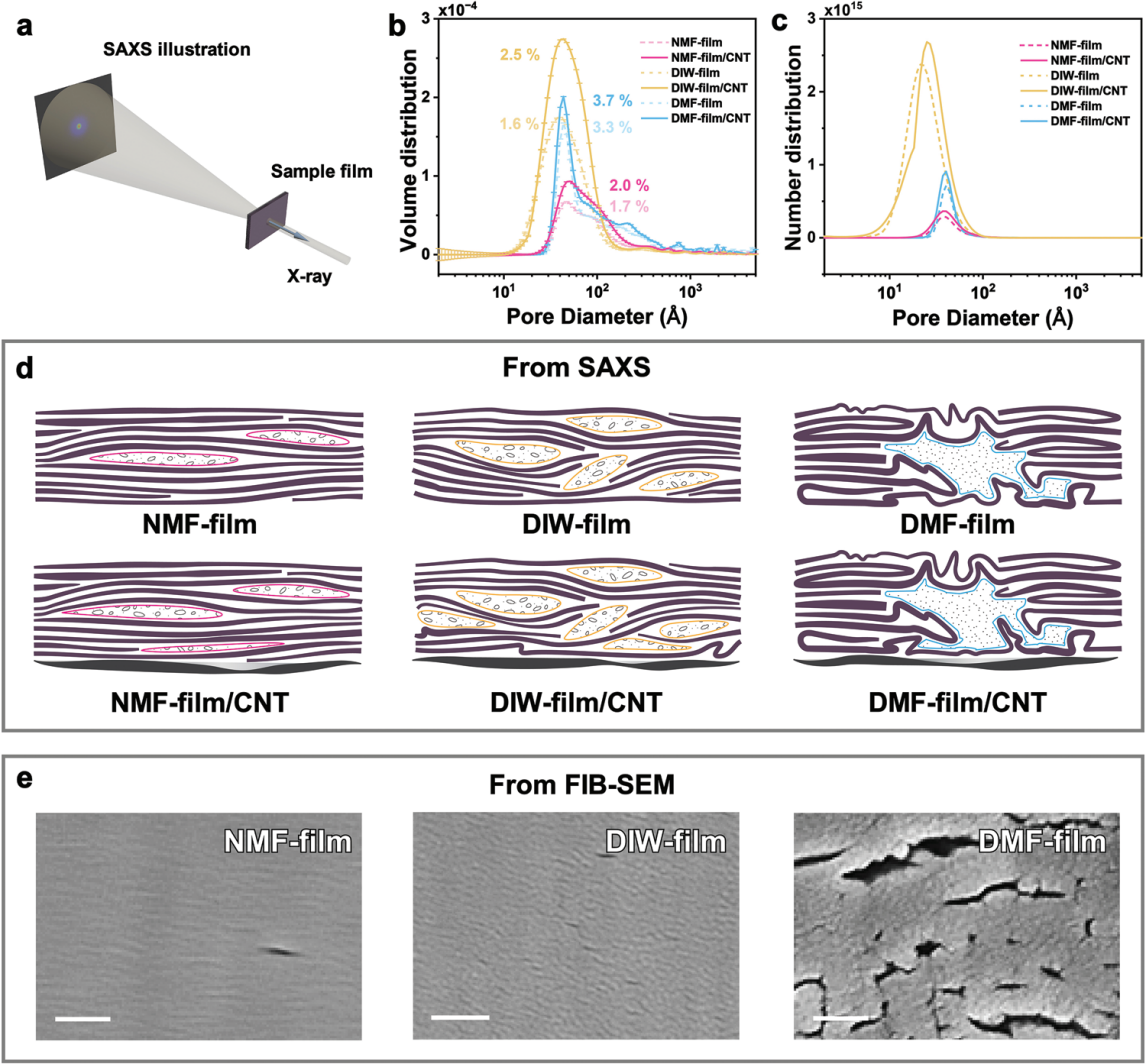
Structural studies of MXene and MXene/CNT Janus films using small‐angle X‐ray methods. a) Schematic illustration of the SAXS measurement setup for the free‐standing films. The blue arrow indicates the *c*‐axis of the lamellar structures of MXenes. b) Pore volume distribution versus pore size in the films fitted from SAXS data. The values indicate the pore volume fraction in the samples. c) Pore number distribution versus pore diameter in the films fitted from SAXS data. The pore diameter is the length of the short axis of the ellipsoid pore with an aspect ratio of 6. d) Structural scheme of MXene flake stacking order in MXene films and MXene/CNT Janus films from SAXS analysis. e) SEM images of cross‐sectional NMF‐film, DIW‐film, and DMF‐film prepared by FIB, scale bar 100 nm.

To investigate the nanometer scale “porous” structures suggested by TEM and SEM, we employed small‐angle X‐ray scattering (SAXS) to obtain the size and fractal information of nanopores, as the setup shown in **Figure** **4**a (see detailed 2D SAXS patterns in Figure S12, Supporting Information). It is worth noting that the porosity is based on our estimation of the scattering contrast. Thus, the value here is used only for comparison among different samples and not as an absolute value. From the SAXS data, we use the interior point gradient /totally non‐negative least squares method to fit the nanopore volume and number distribution^[^ ^43^ ^]^ (Method S3, Supporting Information). The results are plotted in Figure 4b,c. In addition, we employ the unified SAXS model that was fitted to the data, to analyze the pore fractal information (Method S4, Supporting Information).

Based on the WAXS and SAXS modeling above, we can draw a clear scheme for different structures (Figure 4d). NMF‐film is filled with ellipsoidal nanopores with typical pore sizes (minor axis length) from 2 to 20 nm and an amorphous network structure (Figure 4b). Due to the small number of pores, Ti_3_C_2_T_x_ flakes can well‐align along the film plane, and the largest *f* factor is observed. DIW‐film has a similar structure and porosity to NMF film, but includes a higher number of smaller pores, resulting in a worse alignment than NMF film and thus a lower *f* factor. DMF‐film has a very wide distribution of the pore diameter (a few to hundreds of nm), highest porosity, and extremely rough surfaces. Consequently, it has the lowest *f* factor among the three samples. Depositing on CNT substrate will only slightly increase the porosity thus the *f* factor in NMF‐film/CNT and DMF‐film/CNT (24% and 12% respectively). However, due to the poor wetting ability of DIW on CNT, additional pores with larger sizes will appear in DIW‐film/CNT (Figure 4c), which causes a 56% increase in porosity and a discernible decrease of *f*. Such porosity increasing trend agrees with the wetting ability of different solvents on CNT, suggesting the order DMF > NMF > DIW (Figure 2g). We also find a good linear relationship between the decreased *f* and increased porosity (Figure S13, Supporting Information), suggesting that the amorphous pores can randomize the stacking of the MXene flakes. In Figure 4e, the SEM images show very similar patterns with the schematic structures, validating our modeling as a facile and non‐destructive structural investigation tool for MXene stacking structures.

### 2.4 THz Shielding of MXene/CNT Janus Films

**Figure 5:**
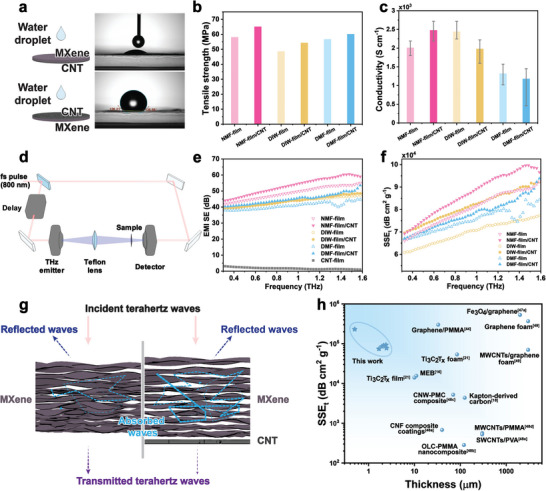
Properties and THz shielding performance of films. a) The water contact angle experiment on the MXene face (top) and CNT face (bottom) of the Janus NMF‐film/CNT. b, c) Comparison of the (b) tensile strength and (c) electrical conductivity of sample films. Error bars represent five independent measurements. d) Schematic illustration of the THz shielding measurement setup. e) Shielding effectiveness of films in the frequency ranging from 0.3–1.6 THz. f) SSE_t_ of sample films after removing the contribution of CNT film. g) Proposed THz shielding mechanism. h) Benchmarking SSE_t_ of this work with previous works.

Potential synergistic mechanical, electrical, and THz EMI shielding properties between MXene and CNT were next explored. As MXenes are vulnerable to water or humid environments,^[^ ^16^ ^]^ the MXene/CNT Janus structure can asymmetrically further provide promoted stability due to the hydrophobicity of CNT layers. This could allow technical benefits in selected asymmetric constructions. **Figure** **5**a shows that a water droplet immediately spreads on the MXene side of the Janus film, i.e., the contact angle is 0^o^ (Movie S1, Supporting Information). In contrast, for the CNT side the contact angle is 136^o^ indicating high hydrophobicity, which can efficiently protect the MXene layer from water intrusion. The CNT layer can also enhance the mechanical performance of the MXene/CNT Janus films compared to pure MXene films, as shown in Figure 5b (for stress–strain curves, see Figure S14, Supporting Information). The NMF‐film/CNT and NMF‐film have higher tensile strength than their DIW and DMF counterparts, which is caused by their lower porosity and better alignment.^[^ ^44^ ^]^

Electrical conductivity is also a crucial factor for THz EMI shielding. As shown in Figure 5c, DIW‐film has a high conductivity (2440 S cm^−1^) among the pure MXene films (DMF‐film ≈1320 S cm^−1^, NMF‐film ≈2010 S cm^−1^), at a similar thickness of 2 µm. Interestingly, after depositing on CNT film, the NMF‐film/CNT shows a 23.2% increase in conductivity, while the conductivity of DIW‐film/CNT and DMF‐film/CNT decreased by 18.9% and 10.6% compared to the pure MXene films. We assume that the better wettability of NMF dispersion on CNT leads to the formation of a tight heterojunction between MXene and CNT film with reduced contact resistance. Thus, the conductivity increased in the NMF‐film/CNT, while decreases in DIW‐film/CNT and DMF‐film/CNT due to non‐negligible contact resistance from their poor heterostructural contact.

The THz shielding performance was explored in the transmission mode with the frequency range of 0.3–1.6 THz, as illustrated in Figure 5d. Completely suppressed and transmitted THz signals in the time domain are measured in sample films and free space (Figure S15a, Supporting Information). As plotted in Figure 5e, NMF‐film with a thickness of 2.0 µm exhibited the highest average THz SE (49 dB), compared to that of DIW‐film (43 dB), and DMF‐film (46 dB) with a similar thickness (also see Figure S15b, Supporting Information). The high SE of NMF‐film may be caused by its low oxidation degree,^[^ ^13^, ^16^ ^]^ thus preventing the degradation of O‐terminated Ti_3_C_2_T_x_ surface with higher THz SE.^[^ ^45^ ^]^ Adding CNT can further increase THz SE in all samples. To better evaluate the THz shielding performance of the MXene layer, we normalized the data by removing the contribution of the CNT layer from the MXene/CNT Janus films and calculated the specific shielding effectiveness (SSE_t_ in dB cm^2^ g^−1^) by dividing the THz SE with sample density per unit area (Figure 5f).^[^ ^44^, ^46^ ^]^ From the normalized data, the average SSE_t_ in NMF‐film (80 211 dB cm^2^ g^−1^) and NMF‐film/CNT (86 866 dB cm^2^ g^−1^) is still higher than the DIW (69 662 dB cm^2^ g^−1^ for DIW‐film and 81 429 dB cm^2^ g^−1^ DIW‐film/CNT) and DMF (74 796 dB cm^2^ g^−1^ for DMF‐film and 78 012 dB cm^2^ g^−1^ DMF‐film/CNT) counterparts. Meanwhile, we also plotted the sample‐to‐sample variations in conductivity and SSE_t_ (Figure S16, Supporting Information). The result confirms that NMF films indeed have higher SSE_t_ than other samples, regardless of the variation of conductivity. The SSE_t_ value shows a similar trend to Figure 5f, where NMF > DIW > DMF; films with CNT > without CNT. This means that even after removing the contribution of CNT film, the Janus films still show a higher SSE_t_ than the pure MXene films. This indicates the structural changes in the MXene layer induced by the CNT substrate have an additional favorable contribution to the THz shielding. Therein, the film porosity was expected to have a role.

We next plotted the porosity ratio versus SSE_t_ ratio of MXene/CNT films to the pure MXene films (Figure S15c, Supporting Information). Interestingly, we find a good linearity, indicating their positive correlation. As proposed in Figure 5g, in the pure MXene films with low porosity, their shielding is dominated by reflection, and the absorption from multi‐reflectance is minor. In the MXene‐CNT Janus film, the new porous structures in the MXene‐CNT Janus film can absorb THz radiation efficiently by multi‐reflectance, thus increasing the overall SE (Figure 5g).^[^ ^47^ ^]^ We also investigate the EMI SE versus MXene loading masses in NMF films (Figure S15d, Supporting Information). Clearly, the shielding performance improves as the weight increases. For a free‐standing film, even ultrathin films are accessible, as the minimum thickness in NMF‐film/CNT is ≈500 nm, where this value is at least 1 µm in pure MXene film. We further compared the SSE_t_ values of our free‐standing Janus MXene/CNT films at 1.4 THz with the reported works (Figure 5h). It is evident that our films show relatively high SSE_t_, with the highest value of ≈2.3 × 10^5^ dB cm^2^ g^−1^. The SSE_t_ is much higher compared to previous work on Ti_3_C_2_T_x_ film and foam^[^ ^18^, ^21^, ^47^, ^48^ ^]^ and comparable with previously reported graphene/PMMA^[^ ^44^ ^]^ graphene foam.^[^ ^49^ ^]^ Our films also show comparable THz SE to MXene with nano‐metamaterial^[^ ^50^ ^]^ and MXene waterborne paint^[^ ^19^ ^]^ (see detailed data in Supporting Table S3) where our thinness allows benefits in the formulation.

## 3 Conclusion

In conclusion, we have identified high permittivity solvents as a superior dispersant for Ti_3_C_2_T_x_ MXene (such as as NMF) and show that they can allow drastically reduced oxidation allowing stability, relevant for all application scenarios, enhanced surface wettability, and structural order in comparison to commonly used polar solvents, i.e., water and DMF. The water sensitivity of MXene was further asymmetrically suppressed by constructing Janus films, i.e., depositing MXene films on hydrophobic CNT substrate by filtration. CNT also enhances the mechanical properties and conductivity in NMF cast film due to the formation of a tight heterostructural interface. The flexible ultrathin MXene/CNT Janus films with low density ≈5.5 mg cm^−2^ show uncommonly high specific THz shielding effectiveness, ranging from 7.7 × 10^4^ to 2.3 × 10^5^ dB cm^2^ g^−1^, where CNT promotes shielding by providing extra “pores” in MXene stackings. Besides, we developed a facile X‐ray scattering model to study the mesoscopic structure of the 2D flake stackings, which can be easily applied to other 2D material stackings. These results are important for the rational structural design of the THz shielding materials using MXene materials. We show that NMF‐processed MXene or MXene/CNT film ranks the best so far (**Table** **2**). We foresee that the findings are general enough to address the stability and wetting problems in MXene and we suggest that high permittivity solvents can be easily applied to disperse other 2D materials, facilitating applications, e.g., energy storage devices, MXene electronics, or nanocomposites.

**Table 2:** Ranking of MXene properties in different solvents.

	Dispersion				Free‐Standing Film			
Samples	Colloidal stability^a^ ^)^	Oxidation Resistance^b^ ^)^	Hydrophilic Surface Wettability	Hydrophobic Surface Wettability	Oxidation ddegree (30 days)^c^ ^)^	Herman's orientation factor	Conductivity [S cm^−1^]	SSE_t_ [dB cm^2^ g^−1^]
NMF	>14 days	>20 days	55.0^o^	31.8^o^	0.34%	0.935	2010	92 017
FA	>14 days	–	–	–	–	–	–	–
DIW	<14 days	<20 days	51.8^o^	136.4^o^	30.81%	0.896	2439	77596
DMF	<14 days	>20 days	27.2^o^	17.7^o^	4.87%	0.878	1319	85 436
NMF‐film/CNT	–	–	–	–	–	0.925	2476	102 413
DIW‐film/CNT	–	–	–	–	–	0.877	1980	93 356
DMF‐film/CNT	–	–	–	–	–	0.871	1179	90 214

^a)^ Time of fully precipitation;

^b)^ Time of forming titanium oxide nanoparticles from TEM;

^c)^ The oxidation degree of films is evaluated by the percentage change of TiO_2‐x_F_2x_ peak area in Ti 2p XPS spectra.

## 4 Experimental Section

### Synthesis of Ti_3_C_2_T_x_ MXene

Ti_3_C_2_T_x_ MXene nanoflakes using the reported method modified from a minimally intensive layer delamination procedure were synthesized.^[^ ^51^ ^]^ Firstly, 2 g of lithium fluoride (LiF, powder, 300 mesh, Sigma–Aldrich Pty. Ltd.) was dissolved in 35 mL of 10 m hydrochloric acid (HCl, ACS reagent, 37% Sigma–Aldrich Pty. Ltd.) by stirring at 35 °C. A suspension of 2 g of Ti_3_AlC_2_ MAX powder (particle size < 40 µm, Carbon‐Ukraine Ltd.) in 5 mL deionized water (DIW) was added dropwise to the above solution. After 24 h, the resulting sediment was washed with DI water until pH reached ≈6. The obtained slurry mainly contains multi‐layered Ti_3_C_2_T_x_ and was then exfoliated by a 1 h vortex in 35 mL of DI water. Finally, the well‐dispersed exfoliated Ti_3_C_2_T_x_ MXene nanoflakes in DIW (DIW‐disp) were obtained by centrifugation at 3500 rpm for 1 h as the supernatant. The as‐prepared DIW‐disp was denoted as a 0‐day sample and stored with N_2_ protection at 4 °C before use. The concentration was measured by weighing a dried film from vacuum filtration of a certain volume dispersion.

Ti_3_C_2_T_x_ MXene NMF/FA/DMF Dispersion

Ti_3_C_2_T_x_ MXene dispersions in NMF, FA, or DMF (NMF/FA/DMF‐disp) were obtained by solvent exchange. Typically, a certain amount of the above DIW‐disp was added into a 50 mL centrifuge tube and centrifugated at 10 000 rpm for 1 h. The light‐colored supernatant was discarded and refilled with target solvents, NMF (99%, Merck Life Science OY), FA (99.5%, Merck Life Science OY), or DMF (99.8%, Merck Life Science OY). After redispersed by vortex, another centrifuge cycle is needed to remove the water residual. Finally, the sediment was re‐dispersed into a certain volume of NMF, FA, or DMF to make a dispersion of ≈2 mg mL^−1^. These fresh‐made samples were regarded as 0‐day dispersions and stored under ambient conditions before use. Dynamic light scattering data and zeta potential of the diluted dispersions were obtained using a Zeta sizer Nano ZS 90.

Preparation of MXene/CNT Janus Films

CNT substrate was prepared using the floating catalyst chemical vapor deposition method by the reported method^[^ ^39^ ^]^ and was collected on a PP membrane (Celgard 3501) without any further treatment. The collection time for a CNT substrate with a thickness of 50 nm and diameter of 3.5 cm was ≈15 min. Transmittance of the CNT substrate was tested with UV–Vis–NIR (Cary 5000).

A simple vacuum filtration method was used to fabricate MXene/CNT Janus films. A certain volume of 1‐day NMF‐disp, DIW‐disp, and DMF‐disp was directly filtrated on the CNT substrate and dried under vacuum overnight at 60 °C. Pure MXene films were prepared with the same method using PP membranes only. After vacuum drying, all the films were stored under ambient conditions. The sample films were labeled according to the different dispersions and the existence of CNT., e.g., a film obtained from NMF‐disp on a CNT substrate is denoted as NMF‐film/CNT. If not specified, all the films are cast from 4 mL of 2 mg mL^−1^ MXene dispersion, which possess a dry weight of ≈8 mg (the weight of the CNT layer can be ignored).

### Chemical Composition Characterization

XPS spectra were performed with a Kratos AXIS Ultra DLD X‐ray photoelectron spectrometer using a monochromated AlKα X‐ray source (1486.7 eV) run at 100 W. A pass energy of 80 eV and a step size of 1.0 eV was used for the survey spectra, while a pass energy of 20 eV and a step size of 0.1 eV was used for the high‐resolution spectra. For data fitting, the binding energies of the high‐resolution spectra were calibrated by setting the C–Ti–O at 282.0 eV.^[^ ^52^ ^]^

### Microscopic Characterization

SEM images were taken with a field‐emission SEM (ZEISS) at an acceleration voltage of 8 kV. The morphologies of MXene nanoflakes and cross‐sectional sample film were verified by JEOL JEM‐2200FS TEM. The sample films were cut by an FIB to provide cross‐sections for SEM and TEM characterization, performed on an FEI Helios NanoLab 600i system. The thickness (*t*) of MXene (/CNT) film was obtained by averaging thickness values from cross‐sectional SEM images cut by FIB at 3–5 different positions. AFM images of Ti_3_C_2_T_x_ flakes on Si substrate were performed using an AFM Dimension Icon scanning probe microscope.

### X‐ray Scattering Measurement and Fitting Methods

XRD measurements of sample films were conducted with a SmartLab (RIGAKU) X‐ray diffractometer operated at 45 kV and 200 mA using CuKα radiation. Small and wide‐angle X‐ray data is obtained using a Xenocs Xeuss 3.0 SAXS/WAXS system (Xenocs SAS, Grenoble, France). The system includes a microfocus X‐ray source (sealed tube) with a Cu target and a multilayer mirror which yields a parallel beam with a nominal wavelength of 1.542 Å (combined Cu K‐α1 and Cu K‐α2 characteristic radiation). The source operates at 50 kV and 0.6 mA. The beam was collimated by a set of variable slits and the beam size at the sample was 0.4 or 0.7 mm during the experiment. The system did not include a beam stop, which enabled direct measurement of sample transmission. The background scattering from the sample holder was normalized and subtracted from the data according to sample transmission. The data was acquired using an area detector (Eiger2 R 1 m, Dectris AG, Switzerland) that was in the evacuated chamber. The sample‐to‐detector distance was calibrated by measuring the diffraction from a known LaB6 standard sample.

### Mechanical, Electrical, and Wetting Tests

Tensile stress–strain curves of MXene (/CNT) strips (20 mm × 5 mm × *t*) were recorded at a loading rate of 1 mm min^−1^ using Instron 5567 tester with a 100 N force cell. The tensile strength was then directly obtained by the maximum stress that the sample film could withstand before breaking. Electron conductivity (σ) was calculated by the thickness (*t*), and sheet resistance (*R*_s_) was measured with an RTS‐8 four‐probe resistivity meter using $\sigma \;=\frac{1}{R_{s}\times t}\;$. Surface hydrophilicity was tested by a contact angle meter (Biolin Scientific, Attention).

### Terahertz Shielding Measurement

The shielding performance of the samples was evaluated at room temperature by a terahertz time‐domain spectroscopy (THz‐TDS) in transmission mode. The time domain terahertz signals were generated with a GaAs photoconductive antenna. A Ti: sapphire laser with 800 nm central wavelength and 50 fs pulse width was used as the excitation source. A silicon‐on‐sapphire photoconductive antenna was used for detection. The working frequency range was 0.3−1.6 THz, and the time step was 0.033 ps.

The SE values were calculated according to the following equation:^[^ ^16^ ^]^

(1) $$SE\;=\;-20\log\;\left(E_{S}/E_{i}\right)$$

where, *E_s_* and *E_i_* are the amplitudes of transmission signals for the samples and the air cavity, respectively.

## Conflict of Interest

The authors declare no conflict of interest.

## Supporting information

Supporting Information

Supplemental Movie 1

## Data Availability

The data that support the findings of this study are available from the corresponding author upon reasonable request.
