# Supplementary material for: High‐permittivity Solvents Increase MXene Stability and Stacking Order Enabling Ultraefficient Terahertz Shielding
Source: Adv Sci (Weinh). 2023 Dec 3;11(5):2305099. doi: 10.1002/advs.202305099 (PMC10837367; doi:10.1002/advs.202305099)
Supplement: Supplementary file 1 — Supporting Information [file ADVS-11-2305099-s001.pdf]

ADVANCED SCIENCE
Open Access

Supporting Information

for *Adv. Sci.*, DOI 10.1002/advs.202305099

High-permittivity Solvents Increase MXene Stability and Stacking Order Enabling Ultraefficient Terahertz Shielding

Xiaodan Hong, Zhenyu Xu, Zhong-Peng Lv, Zhen Lin, Mohsen Ahmadi, Linfan Cui, Ville Liljeström, Volodymyr Dudko, Jiali Sheng, Xiaoqi Cui, Alexey P. Tsapenko, Josef Breu, Zhipei Sun, Qiang Zhang, Esko Kauppinen, Bo Peng* and Olli Ikkala**

SUPPORTING INFORMATION

High-permittivity Solvents Increase MXene Stability and Stacking Order Enabling Ultraefficient Terahertz Shielding

Xiaodan Hong[1], Zhenyu Xu[1], Zhong-Peng Lv[1], Zhen Lin[1], Mohsen Ahmadi[2], Linfan Cui[2], Ville Liljeström[3], Volodymyr Dudko[4], Jiali Sheng[1], Xiaoqi Cui[2], Alexey P. Tsapenko[1,2], Josef Breu[4], Zhipei Sun[2], Qiang Zhang[1,5], Esko Kauppinen[1], Bo Peng[1]*, Olli Ikkala[1]**

[1] Department of Applied Physics, Aalto University, 02150, Espoo, Finland.

[2] Department of Electronics and Nanoengineering, Aalto University, 02150, Espoo, Finland.

[3] Nanomicroscopy Center, OtaNano, Aalto University, 02150, Espoo, Finland.

[4] Bavarian Polymer Institute and Department of Chemistry, University of Bayreuth, D-95447, German.

[5] Honda Research Institute USA, Inc., San Jose, 95134, USA

Corresponding authors:

*Zhong-Peng Lv. Email: zhongpeng.lyu@aalto.fi

*Bo Peng. Email: pengbo006@gmail.com, bo.peng@aalto.fi

*Olli Ikkala. Email: olli.ikkala@aalto.fi

Table of Contents

Supporting Text 2
Figure S1. XRD, AFM, and SEM of $Ti_3C_2T_x$. 9
Figure S2. DLS analysis of $Ti_3C_2T_x$ flakes dispersions in different solvents. 10
Figure S3. SEM images of $Ti_3C_2T_x$ flakes in different solvents. 11
Figure S4. Zeta potential of $Ti_3C_2T_x$ dispersions in different solvents. 12
Figure S5. DLVO plots of $Ti_3C_2T_x$ dispersions in different solvents. 13
Figure S6. TEM and EDS of aged $Ti_3C_2T_x$. 14
Figure S7. XPS survey spectra of 1-day $Ti_3C_2T_x$ films from different solvents. 15
Figure S8. XPS survey spectra of 30-day $Ti_3C_2T_x$ films from different solvents. 16
Figure S9. Characterization of CNT film substrate. 17
Figure S10. SEM cross-sectional images of different films. 18
Figure S11. 2D WAXS patterns of films. 19
Figure S12. 2D SAXS patterns of films. 20
Figure S13. Linear relationship between Herman's orientation factor (*f*) ratios and porosity ratios. ... 21
Figure S14. Tensile-strain test. 22
Figure S15. THz measurement of samples in time-domain, at different frequency and different thickness. 23
Figure S16. Sample-to-sample variations in conductivity and SSEt. 24
Figure S17. The fitting curves *vs* experimental curves of WAXS data using Gauss function. 25
Figure S18. The fitting curves vs experimental curves of SAXS data using size distribution model. . 26
Figure S19. Cross-sectional TEM and statistic aspect ratio of pores. 27
Figure S20. SAXS data fitted using the unified fit model. 28
Table S1. Parameters of $Ti_3C_2T_x$ dispersions in various solvents. 29
Table S2. Calculated Hamaker's constant (A) of $Ti_3C_2T_x$ in various solvents. 29
Table S3. Comparison of THz Shielding performance between previous works and this work. 30

Supporting Text

Supporting Method S1. Colloidal Stability

The negative surface charge of $Ti_3C_2T_x$ MXene is from the dissociation of surface functional groups (−F, −O, −OH). The high permittivity solvent can facilitate the dissociation.[1] The higher solvation energy of ions in NMF and FA than in water also stabilizes the formed ions.[2] Experimentally, this is also proved by an increased conductivity of the MXene dispersion from DMF to NMF and FA. This means that more dissociated ions are formed in NMF and FA than in DIW and DMF.

We can then calculate the concentration of ionic species (c) in different dispersions using $\Lambda = \frac{\kappa}{c}$, where Λ is the limiting molar conductivity and κ is the measured conductivity (Table S1). Then the ionic strength (I) can be calculated using $I = 0.5 \times \sum c_i z_i^2$, where c_i and z_i are the concentration and ion valence number of the ions, respectively. For monovalent salts, $I = c$. We can find that the ionic strength I is one order of magnitude larger in NMF and FA than in DIW, and two orders of magnitude larger than in DMF. Using c and permittivity, we can calculate the Debye length κ^{-1} in different solvents, using the following equation:[3]

$$\kappa^{-1} = \sqrt{\frac{\varepsilon_r \varepsilon_0 RT}{2\times10^3 F^2 c}}. \quad (1)$$

Here, ε_0 is the vacuum permittivity, R is the molar gas constant, T is the temperature, F is the Faraday constant. The obtained κ^{-1} is listed in Table S1.

Based on DLVO theory, the attraction energy (V_A) between two planar surfaces per unit area is:[4]

$$V_A(D) = -\frac{A}{12\pi D^2}, \quad (2)$$

where A is the Hamaker constant, which can be expressed as:[5]

$$A = \frac{3k_B T}{4}\left(\frac{\varepsilon-\varepsilon_S}{\varepsilon+\varepsilon_S}\right)^2 + \frac{3h\omega}{16\sqrt{2}}\frac{(n^2-n_S^2)^2}{(n^2+n_S^2)^{1.5}}, \quad (3)$$

where ε and n are the dielectric constant and refractive index of MXene, respectively. The ε_s and n_s are the dielectric constant and refractive index of solvent medium, respectively. In addition, k_B, h, and T, are the Boltzmann constant, Planck's constant, and temperature (298 K). ω is absorption frequency, and it is 4.73×10^{14} s^{-1} here[6]. The values of A in different solvent are given in Table S2.

The repulsion energy (V_R) between two planar surfaces per unit area is:

$$V_R(D) = 64k_BT\rho_\infty\gamma^2\kappa^{-1}e^{-\frac{D}{\kappa^{-1}}}. \quad (4)$$

ρ_∞ is the number density of ions in the bulk solution, which can be obtained from c, and γ is the dimensionless potential related to the surface potential, expressed as:

$$\gamma = \tanh\left(\frac{ze\psi_0}{4k_BT}\right), \quad (5)$$

where ψ_0 is the potential on the surface. z is the valency of the ion which is 1 here, and e is the elementary charge. Here we also assume γ is a constant for $Ti_3C_2T_x$ dispersed in different solvents, and the value is 50 mV. Thus, the value of γ is −0.46.

We can find V_A is very similar for $Ti_3C_2T_x$ in all solvents, and V_R is related to the ion concentration c and Debye length κ^{-1} in different solvents. We can then plot $V_A(D)$ and $V_R(D)$ in the unit of k_BT nm^{-2}. As shown in **Figure 1c**, the colored dash lines are $V_R(D)$, and the grey dash line is $V_A(D)$. The solid color lines are the total energy $V(D) = V_A(D) + V_R(D)$. We can find the NMF and FA curves have higher energies than those of DIW and DMF. The increasing V with decreasing D in NMF and FA can prevent aggregation. In DIW, the slope is much smaller. In DMF, the energy barrier is flat and small, explaining the easy aggregation of MXene flakes in DMF. The arrow points at Debye length in each solvent. We can find V_R(FA) > V_R(NMF) > V_R(DIW) > V_R(DMF). If we assume the repulsion energy is proportional to the zeta potential, then we can also explain our measured zeta potential (**Figure S4**) where ζ(FA) ≈ ζ(NMF) > ζ(DIW) > ζ(DMF).

We also investigate the potentials $V(D)$ for other organic solvents with low permittivity, e.g., DMAc, DMSO, and NMP. The results are listed in **Table S2** and plotted in **Figure S5**.

Supporting Method S2. WAXS measurement and Herman's orientation factor (*f*)

From WAXS measurement, a 1.5-mm-wide, and 20-mm-long strip was mounted on a homemade 3D printed stage with a sample distance of 5.5 cm, where the film plane was aligned parallel to the beam direction (with the c-axis of the lamellar structure perpendicular to the beam). The azimuthal profiles were obtained from the (002) peak (0.3 Å^{-1} < q < 0.65 Å^{-1}). The numerical values were obtained from a Gauss function that was fitted to the data (**Figure S17**). The maximum value was then set to $\phi = 0$ to facilitate the calculation.

Then, the Herman's orientation factor (*f*) was calculated from the obtained Gauss function using the given equation:[7]

$$f = \frac{1}{2}\left(\frac{3\int_0^{\frac{\pi}{2}} I(\phi)cos^2\phi sin d\phi}{\int_0^{\frac{\pi}{2}} I(\phi) sin d\phi} - 1\right), \quad (6)$$

where the $I(\phi)$ is the intensity at an azimuthal angle of ϕ. The full width at half maximum (FWHM) of the azimuthal peak can be directly obtained from the Gauss function as another indicator for the alignment degree.

Supporting Method S3. SAXS measurement and porosity fitting

For SAXS measurements related to porosity, 5 × 5 mm samples were mounted perpendicular to the beam direction with a sample-to-detector distance of 110 cm. In this configuration the *c*-axis of the lamellae is parallel to the beam. The azimuthally averaged 1D SAXS curve (0.012 $Å^{-1} < q < 0.2$ $Å^{-1}$) from the isotropic 2D pattern was used for pore size distribution fitting with Internal Point Gradient / Total Non-Negative Least Square (IPG/TNNLS) Method (**Figure S18**) in Irena:[8]

$$I(q) = |\Delta\rho^2| \int |F(q,r)|^2 \left(V(r)\right)^2 N_p(r) dr, \quad (7)$$

where $I(q)$ is the experimental intensity, $F(q,r)$ is the form factor regarding the pore diameter, $V(r)$ is the scattering volume of the pore with diameter r, and N_p(r) is the histogram size distribution. For $F(q,r)$, an aspect ratio of 6 of ellipsoid pores was used in IPG/TNNLS fitting, which was estimated from cross-sectional TEM analysis (**Figure S19**). Here, we use an r range from 2–5000 Å with 100 logarithmic bins for N_p(r). We assume that these nanopores include amorphous or polycrystalline TiO_2 as well as polycrystalline $Ti_3C_2T_x$, which forms a network structure with different electron densities than crystalline $Ti_3C_2T_x$. We estimate the contrast $|\Delta\rho^2|$ between crystalline $Ti_3C_2T_x$ and polycrystalline or amorphous pore is 1×10^{20} cm^{-4}. For the fitting, a flat background was used, and all fittings meet the criteria of iterations > 1000 and chi-square < 677.

Supporting Method S4. Unified model for fitting pore fractal

Unified model in Irena[9] was employed to fit the 1D SAXS curves, where the radius of gyration (R_g) of the nanopores as well as the power-law slope (P) for pore fractal information can be obtained.[10] Three structural levels (high-q, middle-q, and low-q) were used, and each level has a Guinier region followed by a power-law region. For high-q (level 1) and middle-q (level 2) regimes, the coefficient of the power-law region is estimated from the coefficient of the Guinier region, R_g and P, to simplify the fitting parameters. Low-q regime (level 3) only has power-law region from scattering of macrostructures, which is not discussed here due to the limited q range of our measurement.

In the high-q regime, structures with R_{g1} = 4 Å were used to fit the “knee-like” feature for all samples (**Figure S20**) with the power-law P_1 = 4 (smooth surface). These structures mainly represent the density variation at the atomic scale. For example, the interlayer spacing between $Ti_3C_2T_x$ flakes, where the intercalant molecules of a few Å size (H_2O ~ 3 Å, NMF/DMF ~ 4 Å) with lower electron density are present.

The middle-q regime (level 2), where the nanopores formed by amorphous region, contribute to the major part of the experimental curves. In NMF-film/CNT and NMF-film (**Figure S20a**), the unified fitting results show an R_{g2} of 60 Å, which represent an ellipsoid pore with 44 Å diameter in the short axes and 264 Å in the long axis based on our assumption. The pore size from unified fitting well-agrees with the pore size distribution using IPG/TNNLS Method. We can also get a power-law P_2 = 2.9 from the fitting. In pore fractal, where $I(q) \propto q^{-D_p}$, a D_p value of 2.9 represents the nanopores have a disordered network of small microstructures.[11] In DIW-film/CNT and DIW-film (**Figure S20b**), the fitting gives an R_{g2} of 45 Å and P_2 of 2.7, meaning that the nanopore size is smaller than those in NMF samples, but the pore fractal is similar. The disordered network in the nanopores of NMF and DIW samples

again proves the amorphous nature of their structure. We assume that in these nanopores, there are amorphous or polycrystalline TiO_2, as well as polycrystalline $Ti_3C_2T_x$, which forms a network structure with different electron densities than crystalline $Ti_3C_2T_x$.

However, we found a much larger pore size and different fractal structure in DMF-film/CNT and DMF-film. As shown in **Figure S20c**, the R_{g2} value is 140 Å, much larger than films cast from NMF and DIW, consistent with the IPG/TNNLS fitting results. In films cast from DMF, we have a P_2 = 3.2, which should be applied to the surface fractal, where $I(q) \propto q^{D_s-6}$ and D_s = 2.8.[12] In this case, there are mainly larger pores with an extremely rough surface in DMF-film/CNT and DMF-film.

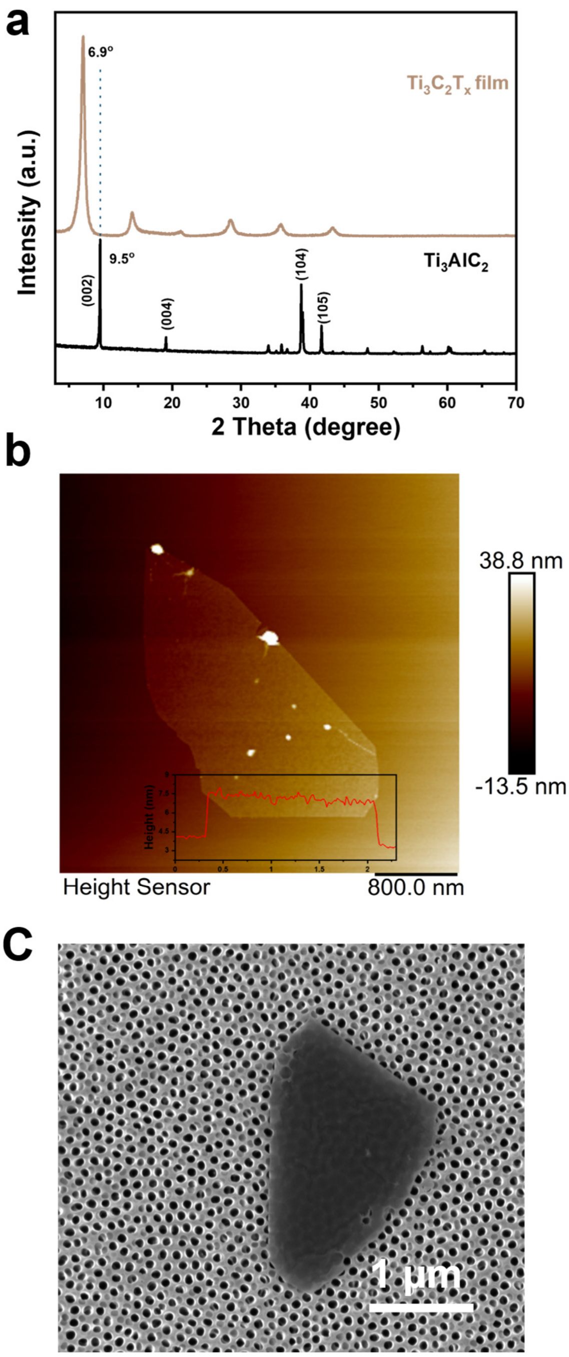

Figure S1. XRD, AFM, and SEM of $Ti_3C_2T_x$.

(**a**) XRD patterns of Ti_3AlC_2, and $Ti_3C_2T_x$ MXene films, (**b**) AFM image and height profile (inset) of $Ti_3C_2T_x$ flake, (**c**) SEM image of few-layered $Ti_3C_2T_x$ on the AAO substrate.

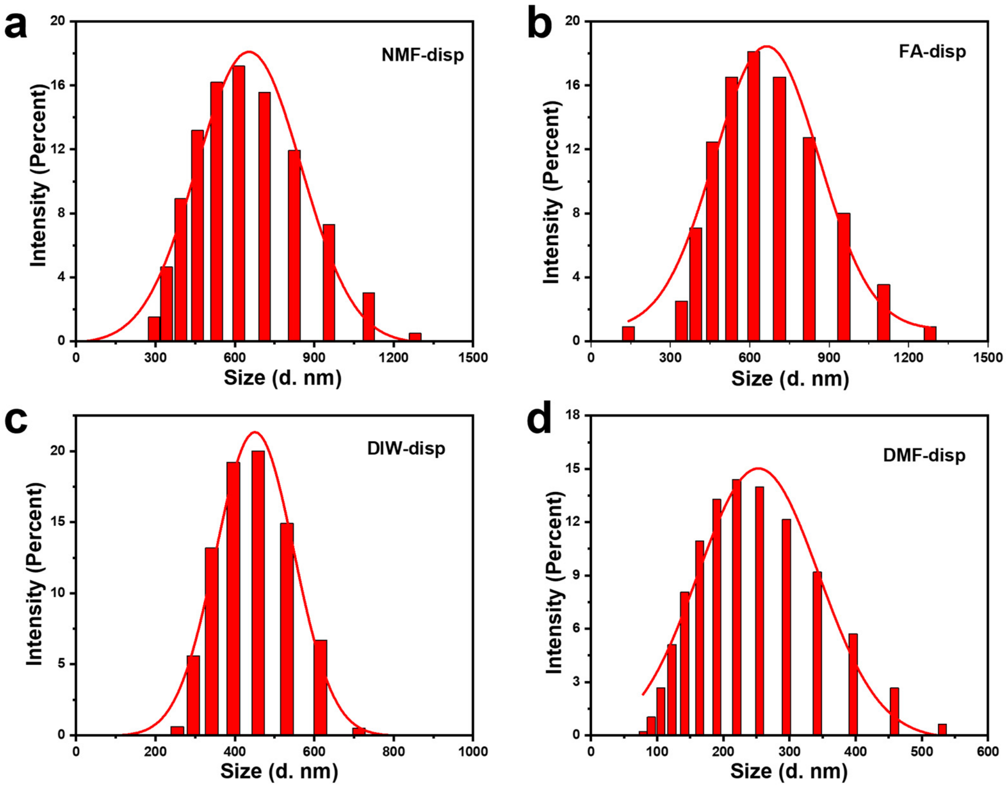

Figure S2. DLS analysis of $Ti_3C_2T_x$ flakes dispersions in different solvents.

DLS analysis of $Ti_3C_2T_x$ flakes dispersions in (**a**) NMF, (**b**) FA, (**c**) DIW, and (**d**) DMF.

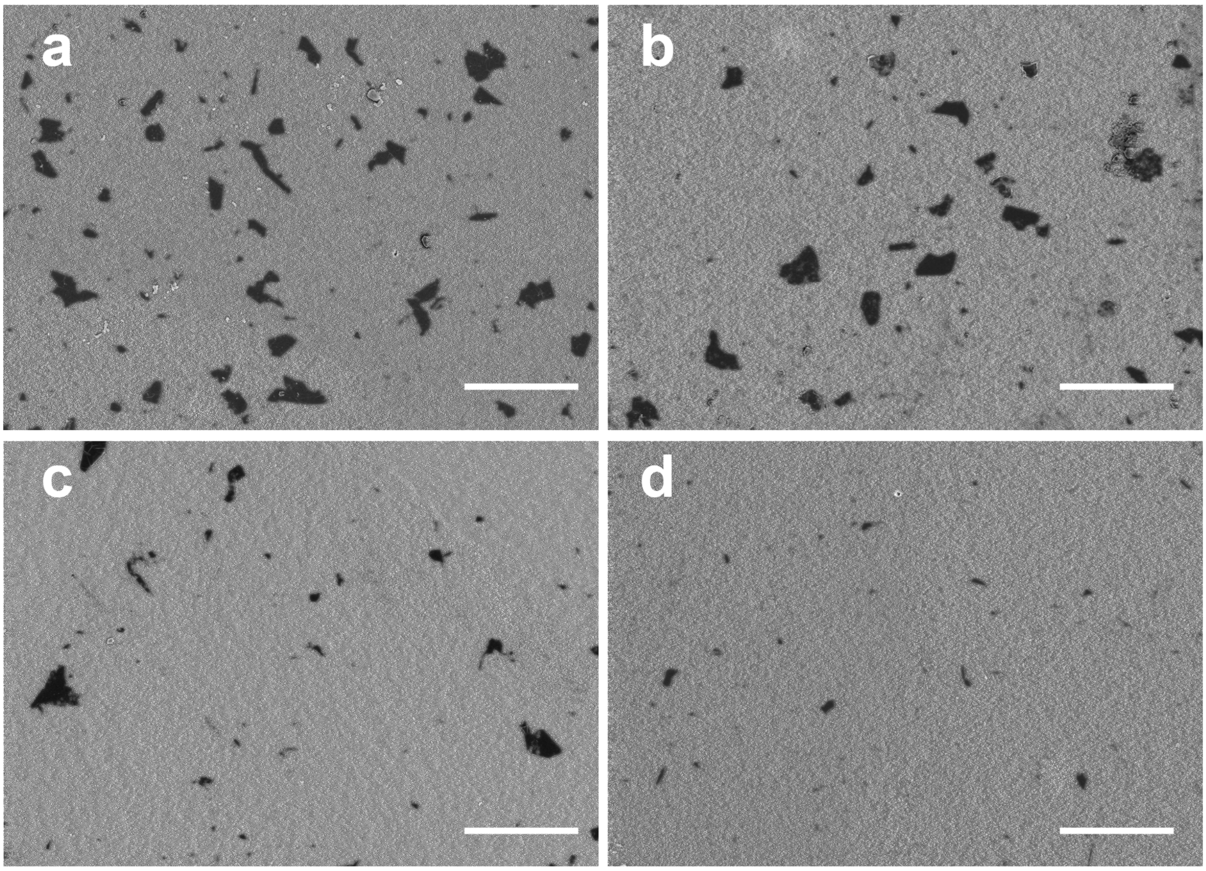

Figure S3. SEM images of $Ti_3C_2T_x$ flakes in different solvents.

SEM images of few-layered $Ti_3C_2T_x$ in (**a**) NMF, (**b**) FA, (**c**) DIW, and (**d**) DMF on the AAO substrate, scale bars: 10 μm.

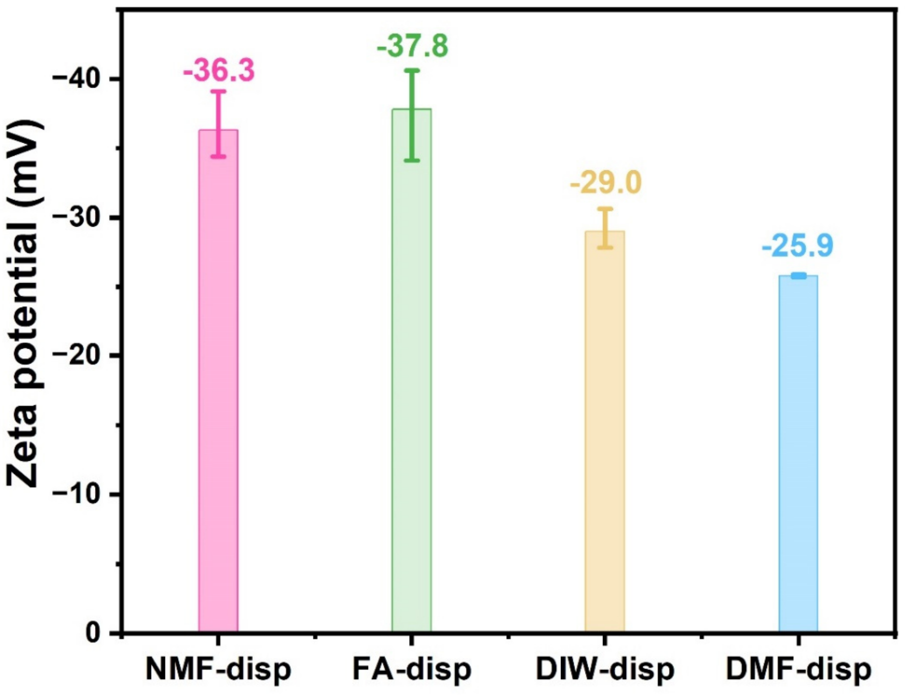

Figure S4. Zeta potential of $Ti_3C_2T_x$ dispersions in different solvents.

Zeta potential of $Ti_3C_2T_x$ dispersions in NMF, FA, DIW, and DMF solvents.

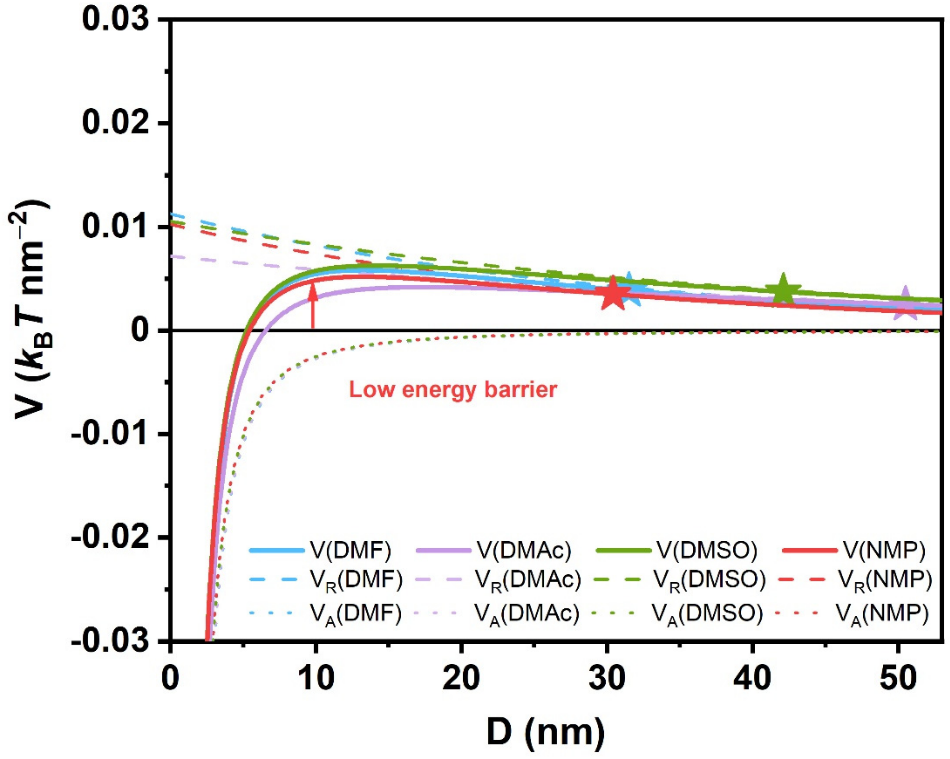

Figure S5. DLVO plots of $Ti_3C_2T_x$ dispersions in different solvents.

Schematic DLVO plots using planar charged surfaces model. The repulsion energy V_R (color dash curves) is calculated from experimental data (see Supporting Method S1). The star symbols mark the Debye lengths κ^{-1} on V_R curves.

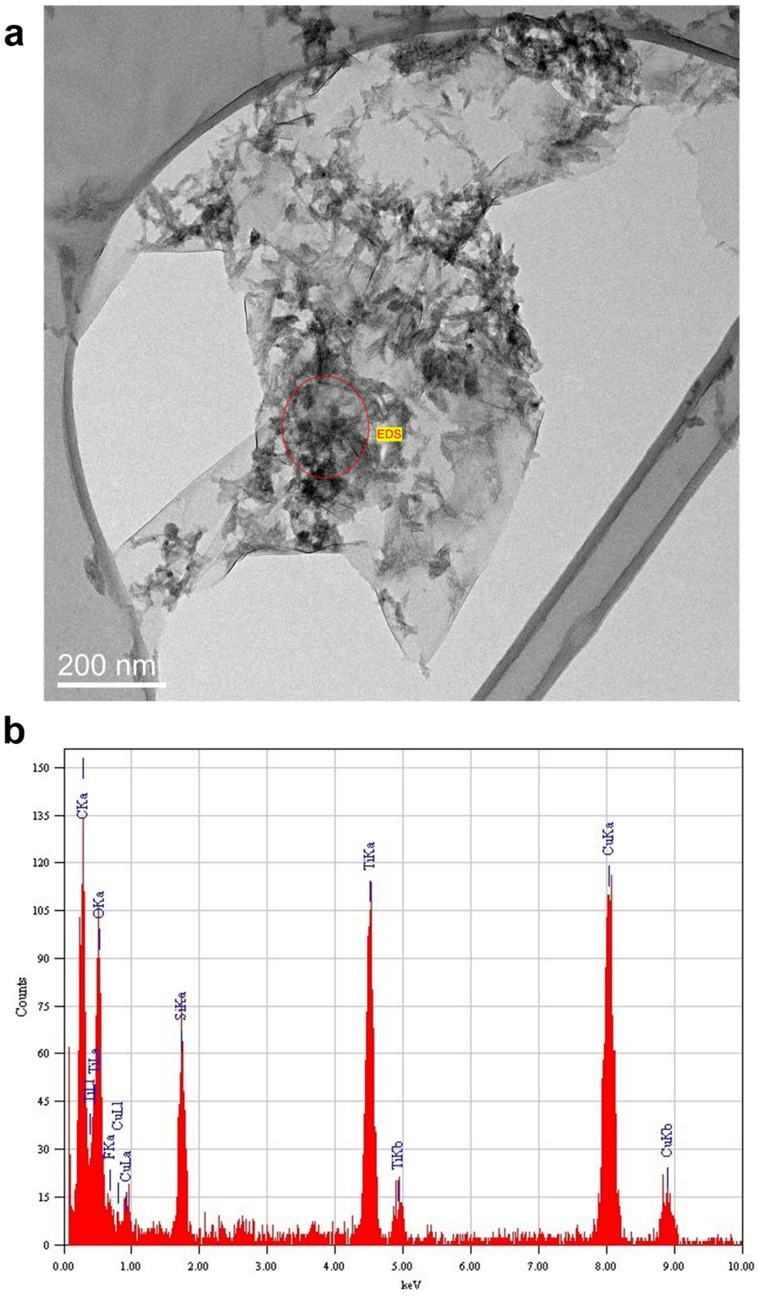

Figure S6. TEM and EDS of aged $Ti_3C_2T_x$.

(**a**) TEM image of aged $Ti_3C_2T_x$ flakes in DIW, and (**b**) EDS results of a selected area.

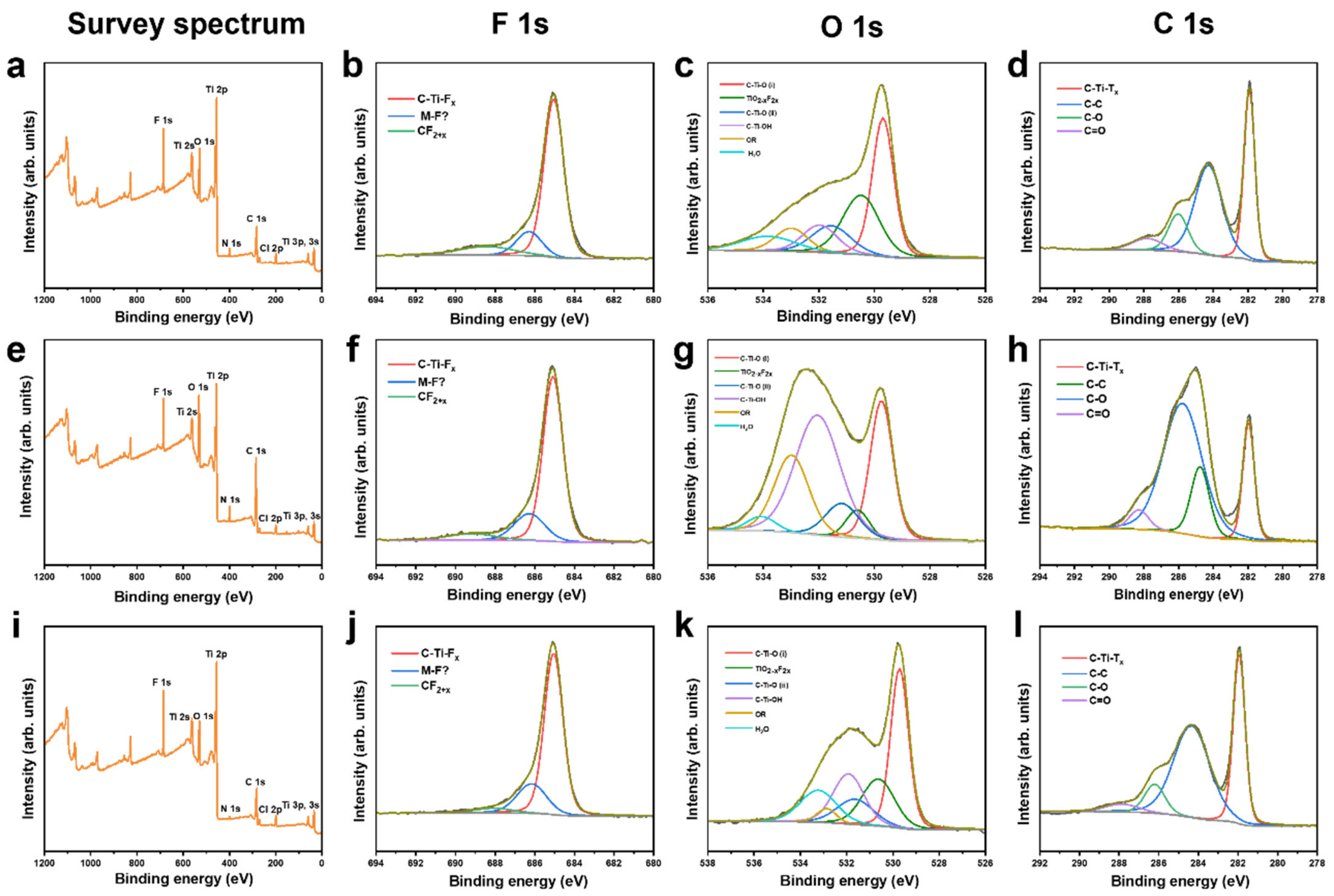

Figure S7. XPS survey spectra of 1-day $Ti_3C_2T_x$ films from different solvents.

XPS survey spectra of 1-day (**a** to **d**) NMF-film, (**e** to **h**) DIW-film, and (**i** to **l**) DMF-film, and corresponding high resolution XPS spectrum of F1s, O1s, and C1s.

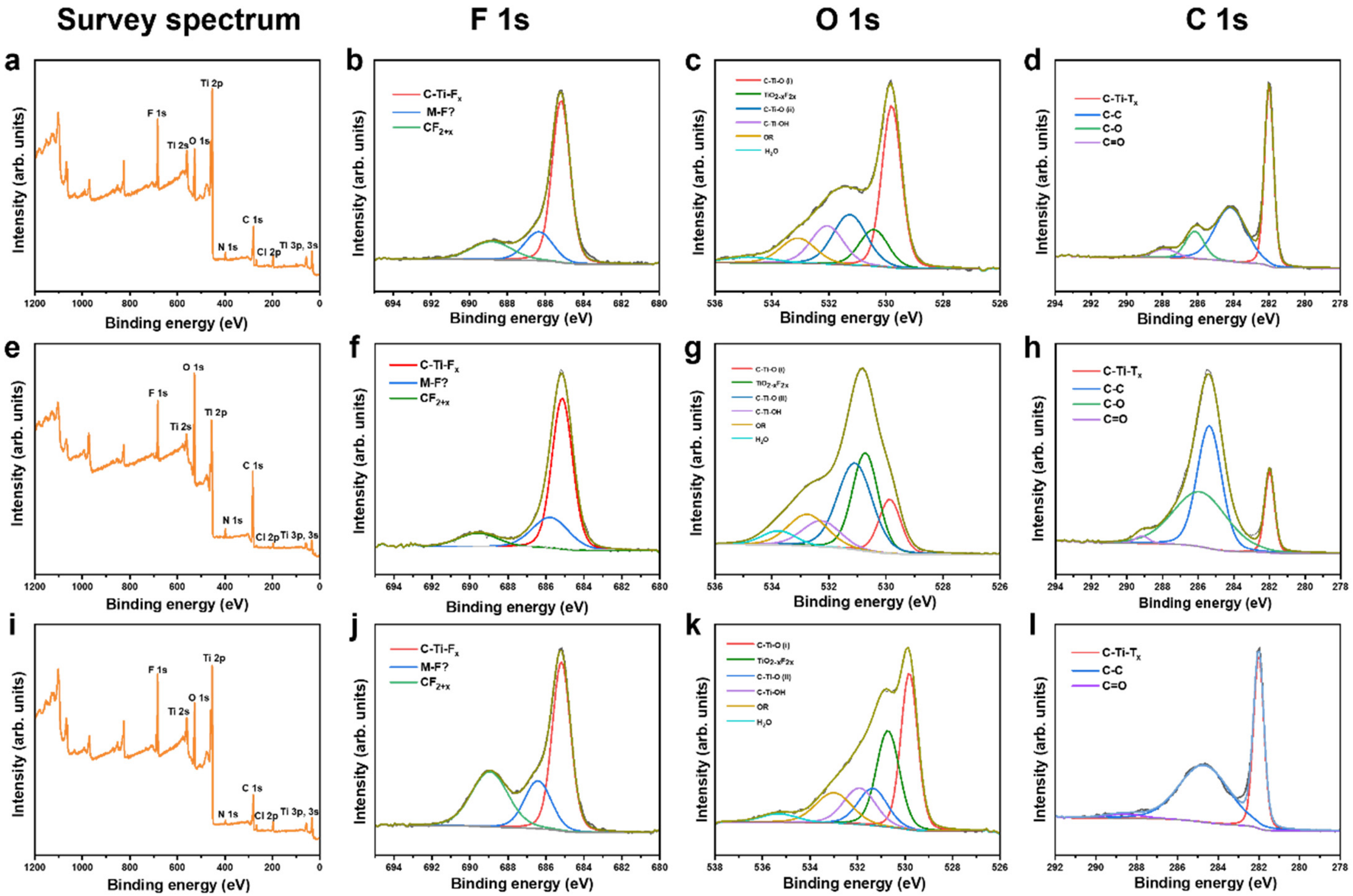

Figure S8. XPS survey spectra of 30-day $Ti_3C_2T_x$ films from different solvents.

XPS survey spectra of 30-day (**a** to **d**) NMF-film, (**e** to **h**) DIW-film, and (**i** to **l**) DMF-film, and corresponding high-resolution XPS spectrum of F1s, O1s, and C1s.

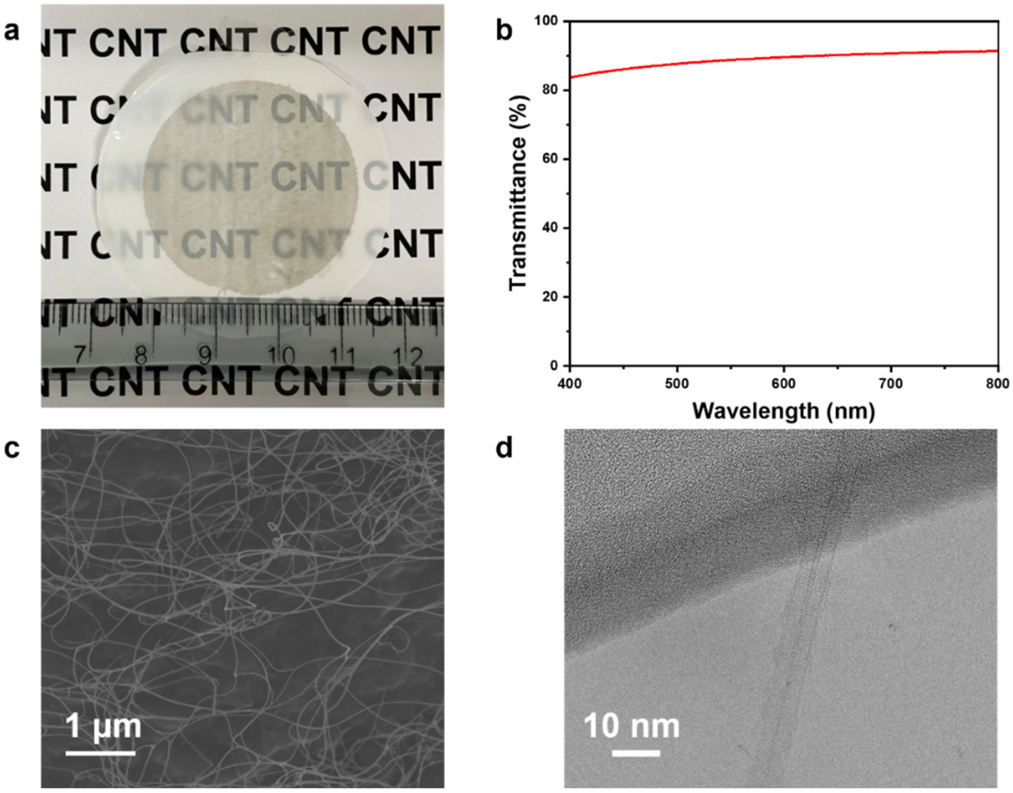

Figure S9. Characterization of CNT film substrate.

(**a**) Digital photo of CNT film deposited on a Celgard 3501 PP membrane, (**b**) UV-Vis spectra of the CNT film, (**c**) SEM, and (**d**) TEM images of CNTs.

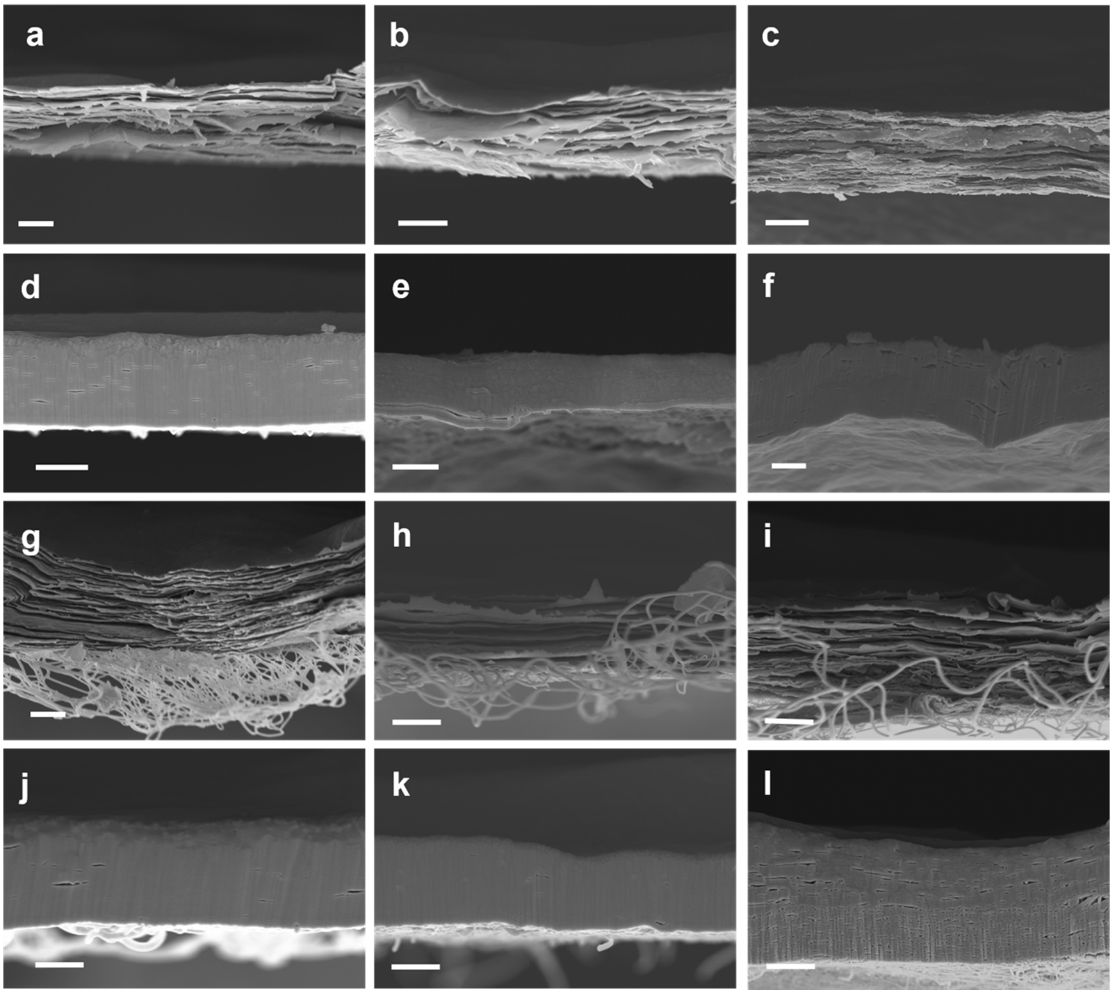

Figure S10. SEM cross-sectional images of different films.

SEM cross-sectional images of (**a**) NMF-film, (**b**) DIW-film, (**c**) DMF-film, and (**g**) NMF-film/CNT, (**h**) DIW-film/CNT, (**i**) DMF-film/CNT. SEM images of cross-sections cut by FIB for (**d**) NMF-film, (**e**) DIW-film, (**f**) DMF-film, and (**j**) NMF-film/CNT, (**k**) DIW-film/CNT, (**l**) DMF-film/CNT. Scale bars: 1 μm.

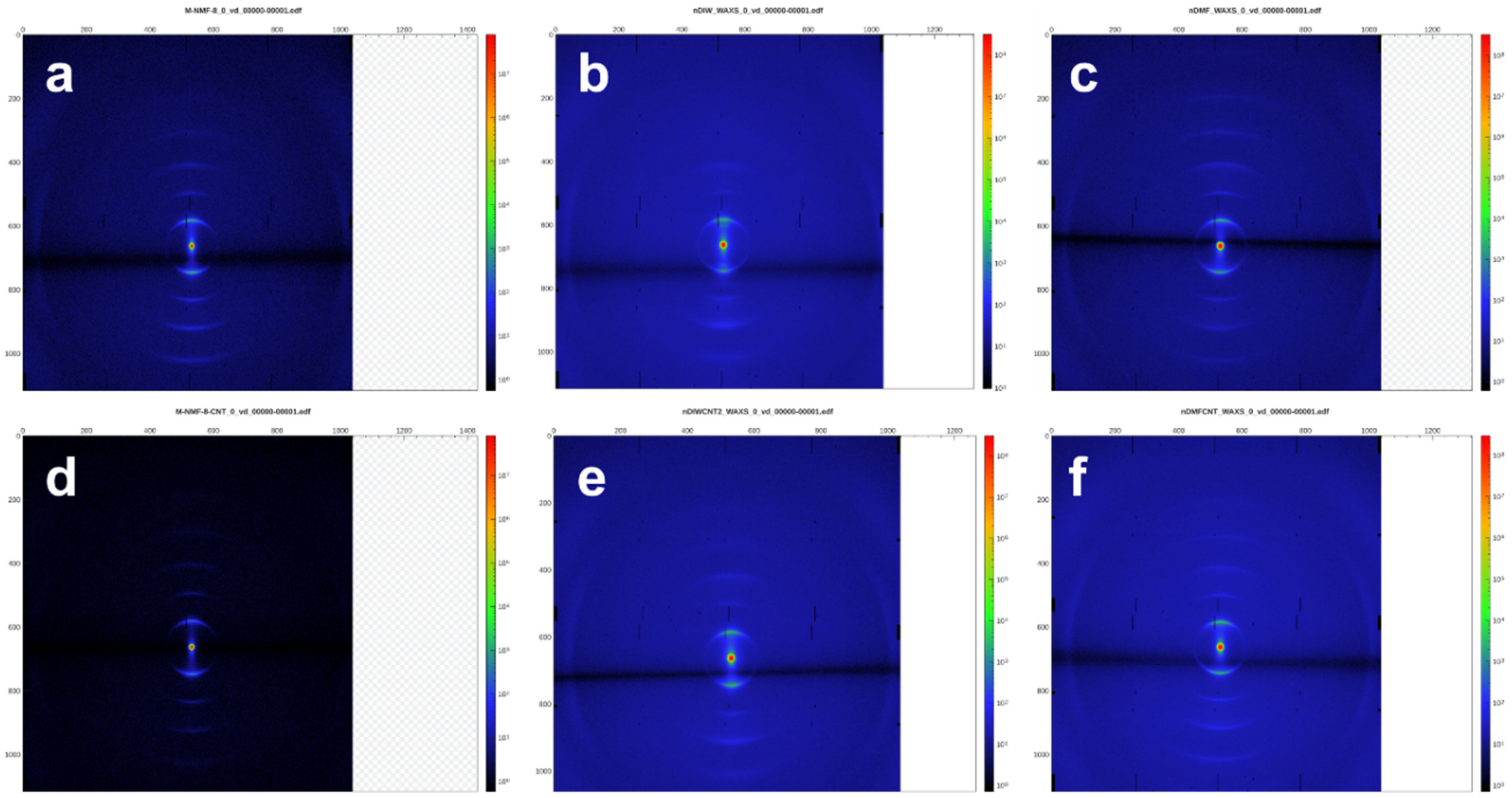

Figure S11. 2D WAXS patterns of films.

WAXS patterns for an incident Cu-Kα X-ray beam parallel to the sample film plane for (**a**) NMF-film, (**b**) DIW-film, (**c**) DMF-film, and (**d**) NMF-film/CNT, (**e**) DIW-film/CNT, (**f**) DMF-film/CNT.

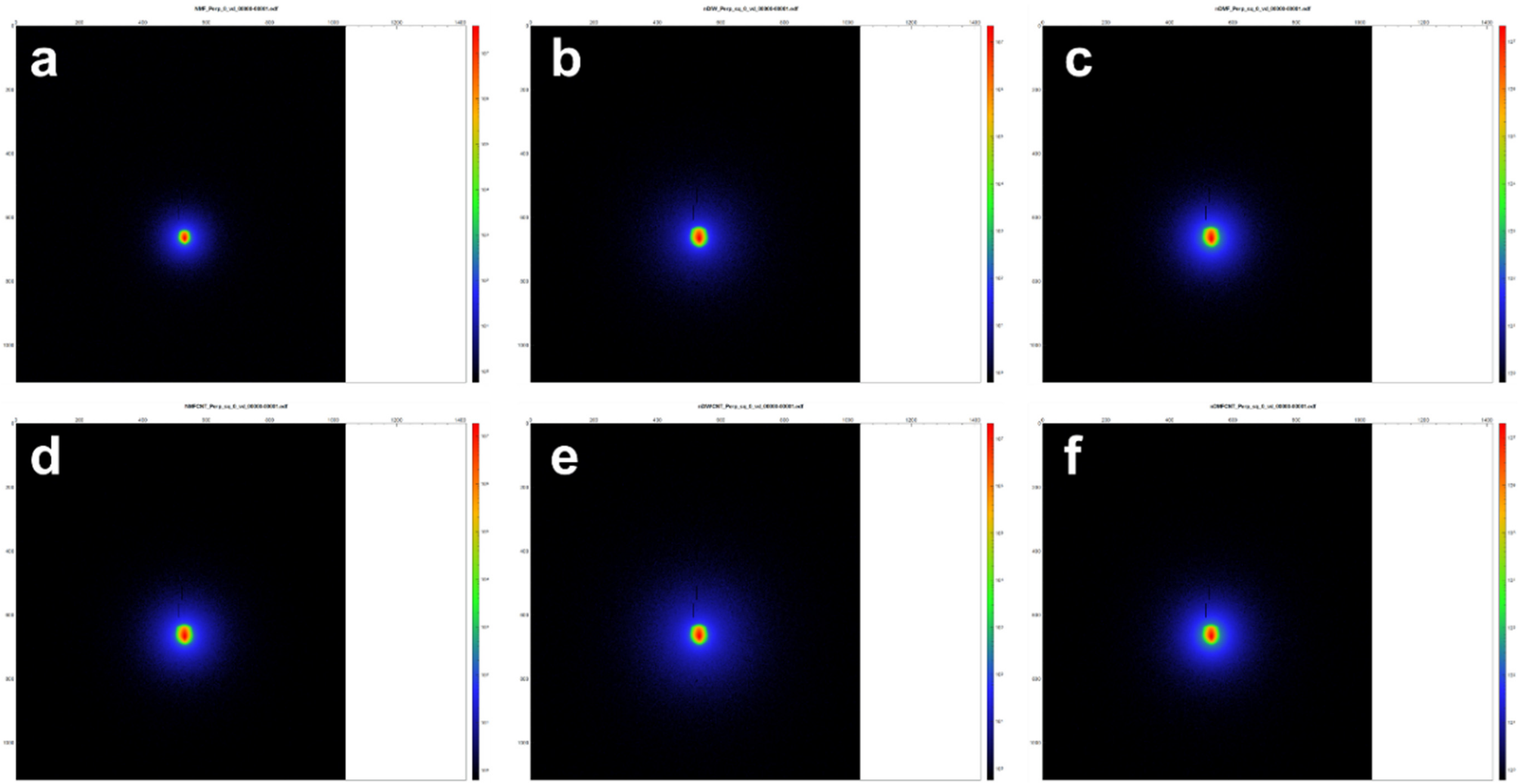

Figure S12. 2D SAXS patterns of films.

SAXS patterns for an incident Cu-Kα X-ray beam perpendicular to the sample film plane for (**a**) NMF-film, (**b**) DIW-film, (**c**) DMF-film, and (**d**) NMF-film/CNT, (**e**) DIW-film/CNT, (**f**) DMF-film/CNT.

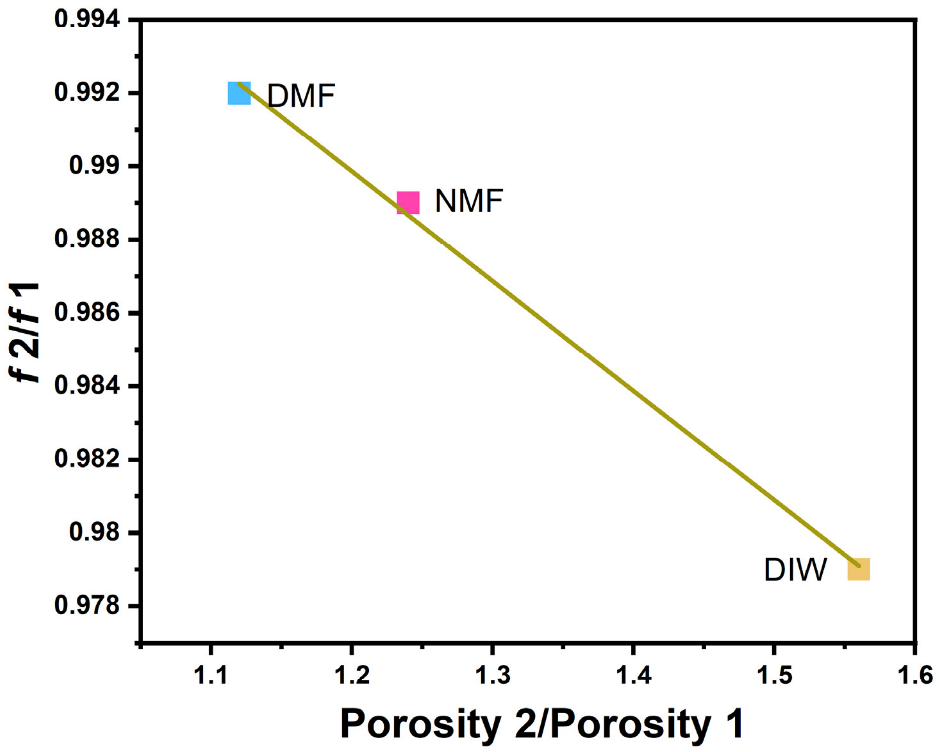

Figure S13. Linear relationship between Herman's orientation factor (*f*) ratios and porosity ratios.

f 2 and porosity 2 means the *f* factor and porosity of MXene/CNT films, 1 represents that of pure MXene films.

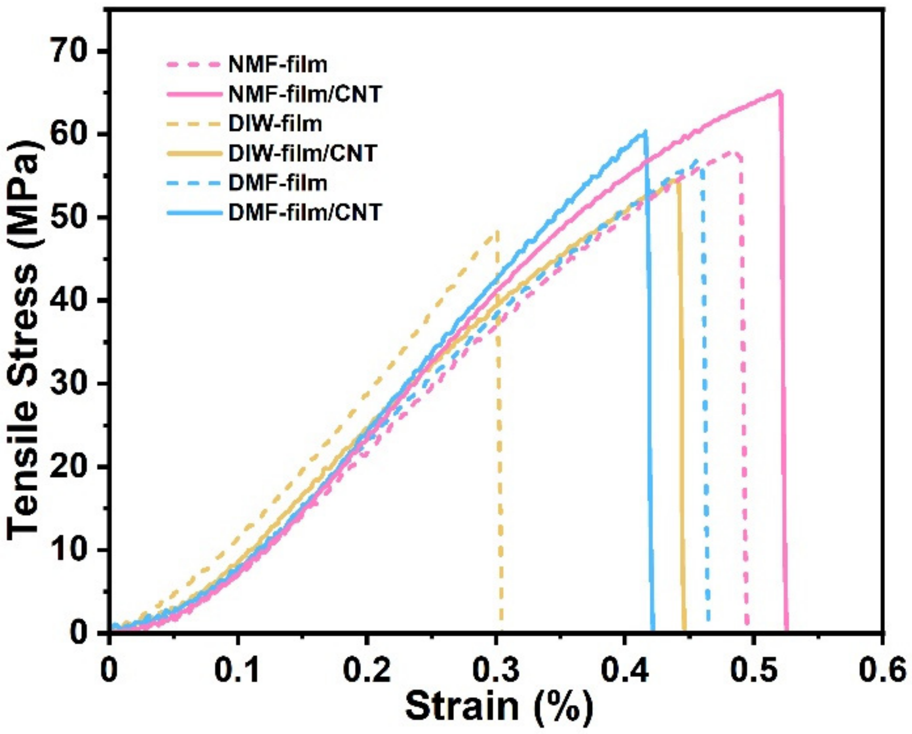

Figure S14. Tensile-strain test.

Tensile-strain curves of MXene films without and with CNT substrates.

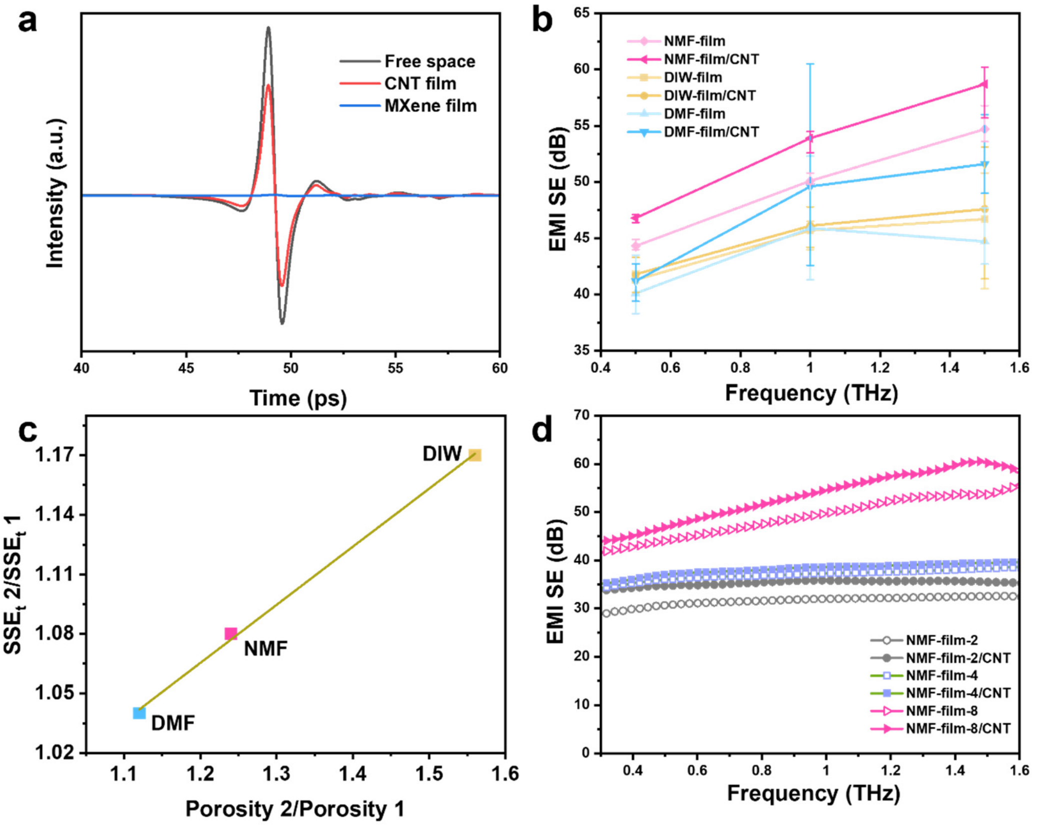

Figure S15. THz measurement of samples in time-domain, at different frequency and different thickness.

(**a**) transmission time-domain spectra of free space, CNT film, and MXene film, (**b**) average EMI Shielding Effectiveness at 0.5 THz, 1 THz, and 1.5 THz of three batches of sample films, (**c**) The scatter graph of the relationship between SSE_t and porosity. SSE_t 2 and porosity 2 mean the SSE_t and porosity of MXene/CNT films, and 1 represents that of pure MXene films. (**d**) EMI Shielding Effectiveness of NMF-film-2/4/8 and NMF-film-2/4/8/CNT with different MXene loading mass, where 2, 4, and 8 represent the theoretical loading mass of MXene flakes.

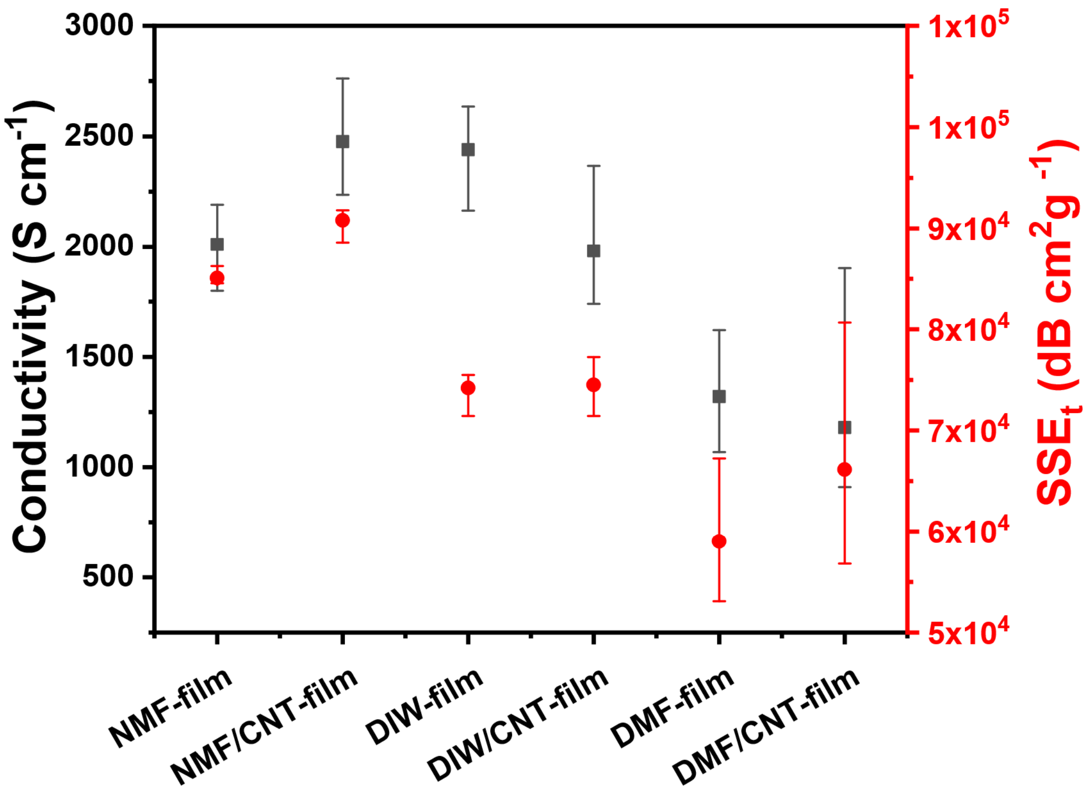

Figure S16. Sample-to-sample variations in conductivity and SSEt.

Sample-to-sample variations in conductivity and SSEt with error bars. Error bars represent five independent measurements.

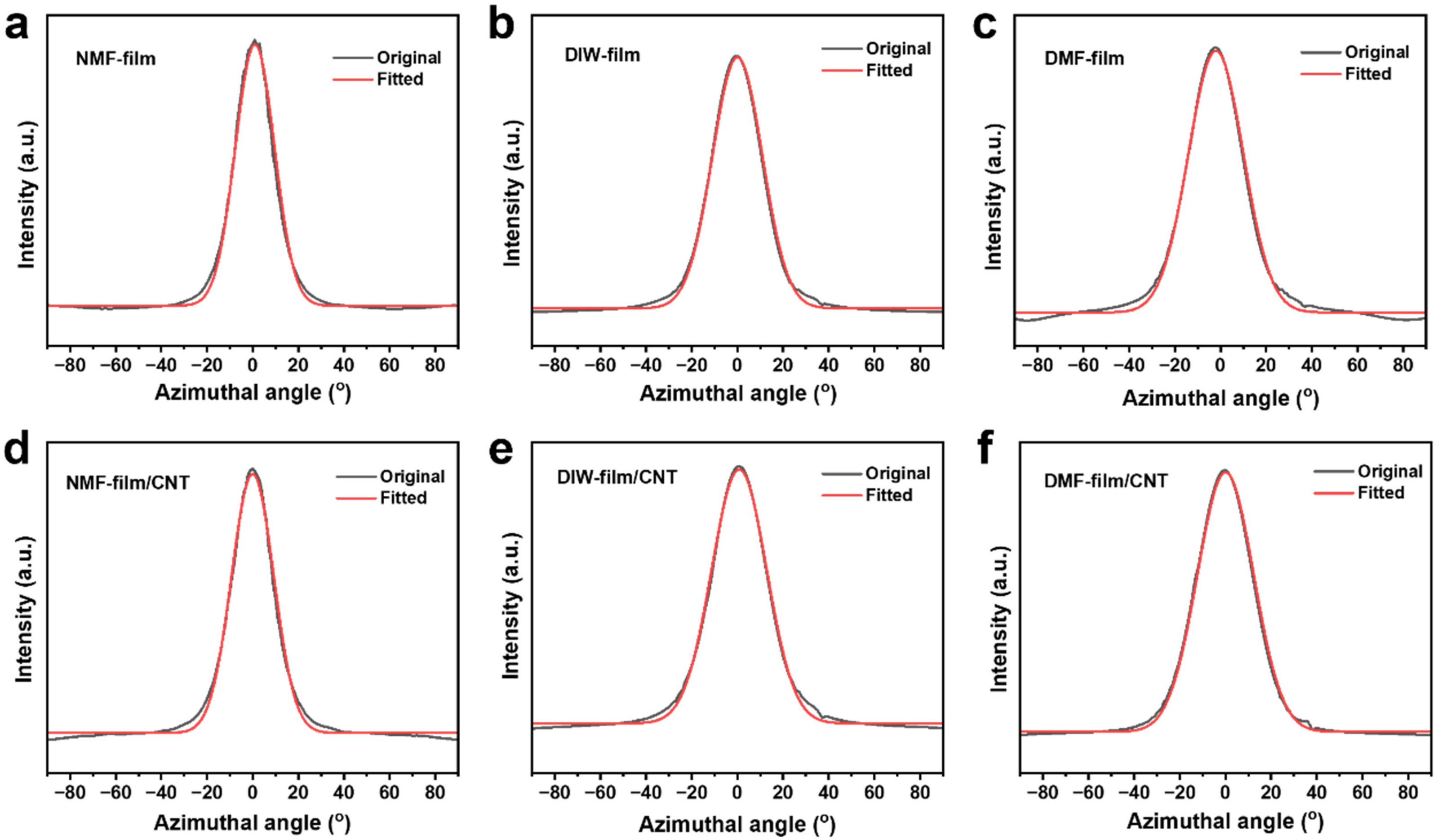

Figure S17. The fitting curves *vs* experimental curves of WAXS data using Gauss function.

(**a**) NMF-film, (**b**) DIW-film, (**c**) DMF-film, (**d**) NMF-film/CNT, (**e**) DIW-film/CNT, and (**f**) DMF-film/CNT.

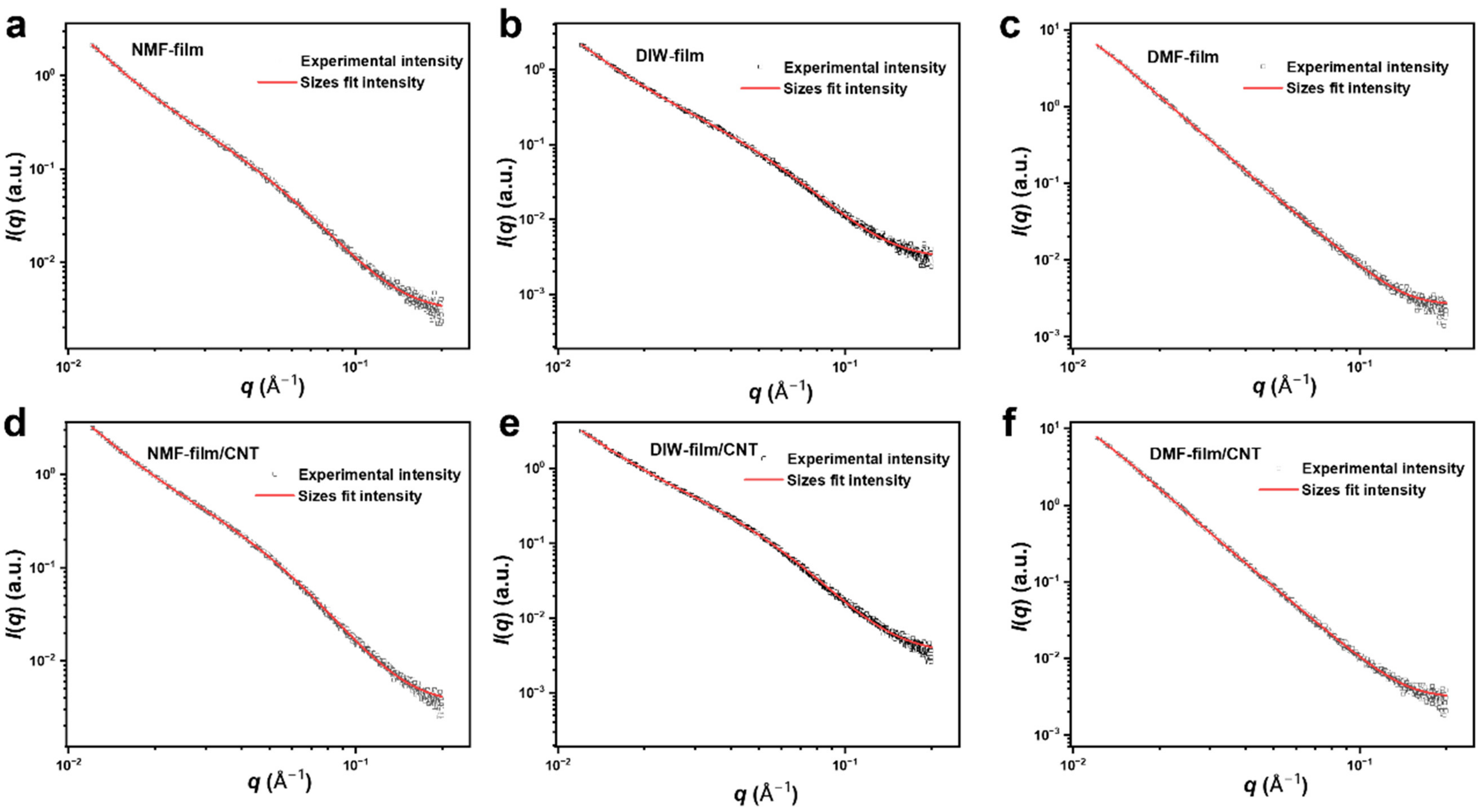

Figure S18. The fitting curves vs experimental curves of SAXS data using size distribution model.

(**a**) NMF-film, (**b**) DIW-film, (**c**) DMF-film, (**d**) NMF-film/CNT, (**e**) DIW-film/CNT, and (**f**) DMF-film/CNT.

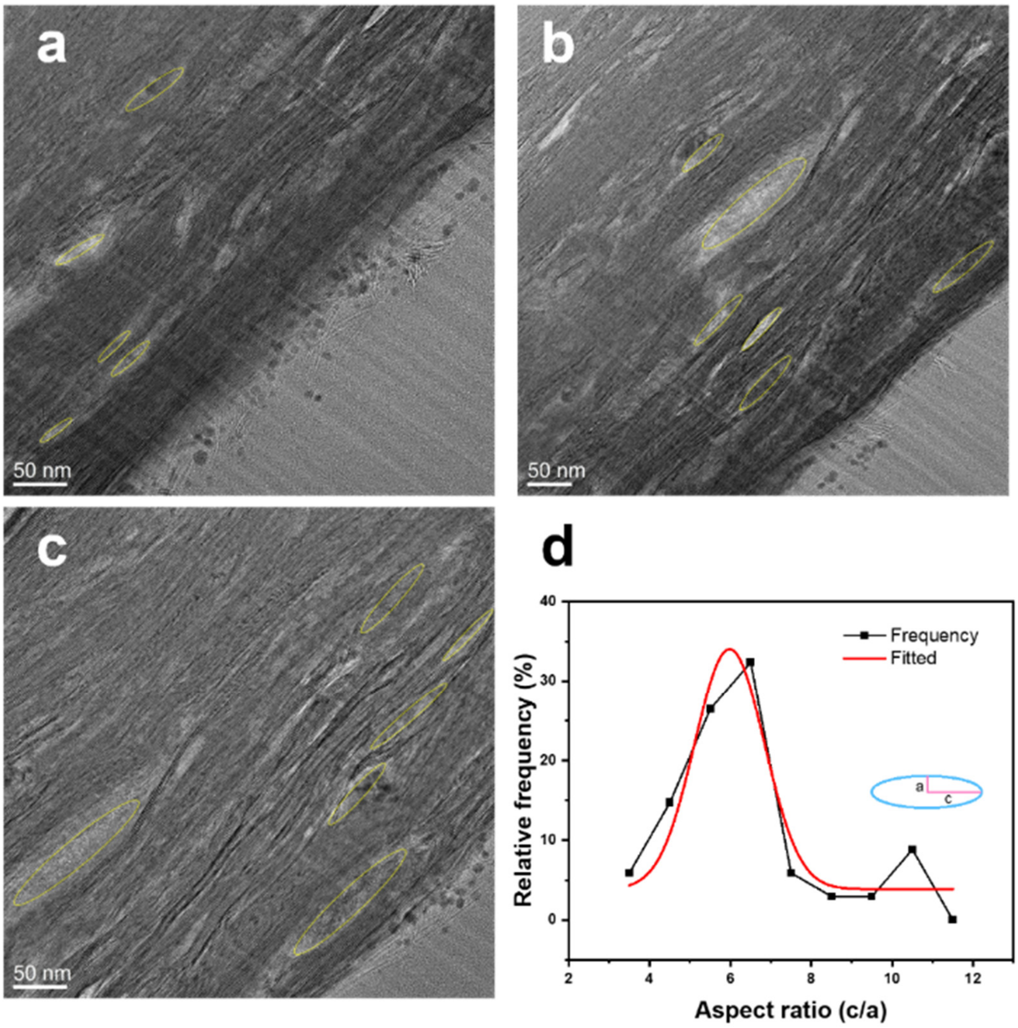

Figure S19. Cross-sectional TEM and statistic aspect ratio of pores.

(**a** to **c**) TEM images of cross-sectional NMF-film/CNT Janus free-standing film in different positions prepared by FIB. (**d**) Statistic aspect ratio of marked ellipsoid pores in panels a–c. Gauss function was used for fitting the mean aspect ratio.

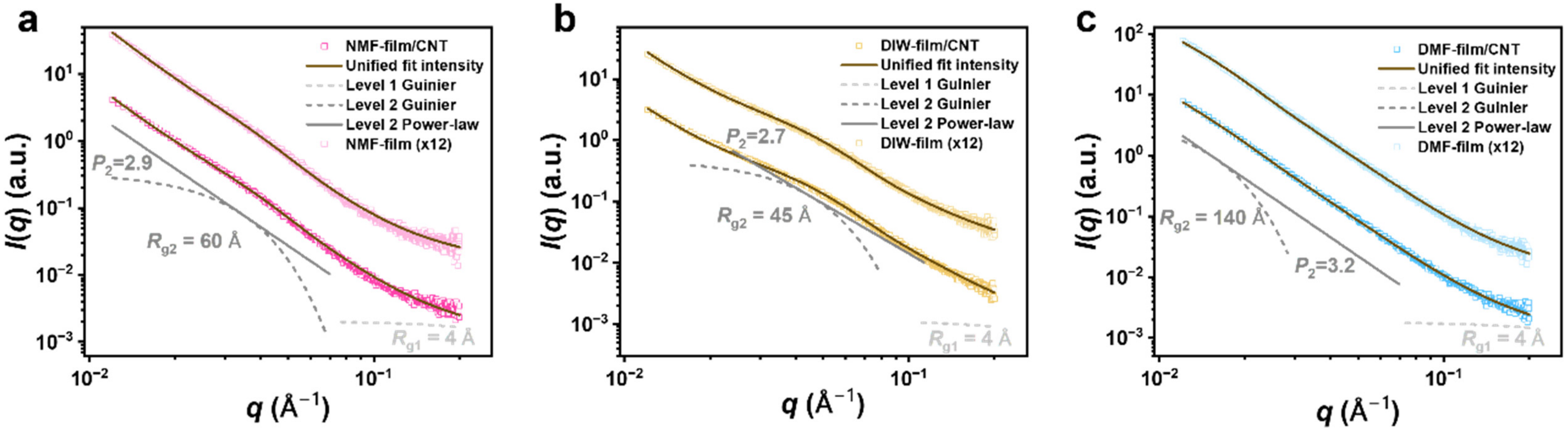

Figure S20. SAXS data fitted using the unified fit model.

(**a**) NMF-film and NMF-film/CNT (**b**) DIW-film and DIW-film/CNT, and (**c**) DMF-film and DMF-film/CNT.

Table S1. Parameters of $Ti_3C_2T_x$ dispersions in various solvents.

Properties	NMF	FA	DIW	DMF	DMAc	DMSO	NMP
Conductivity κ at 0.05 mg/mL(mS/cm)	0.0656	0.157	0.0484	0.00377	0.00107	0.00102	0.00137
Λ^0_{salt} (S cm^2 mol^{-1},298K)	50[a)]	29[b)]	120[c)]	85[d)]	61[e)]	33[f)]	33[g)]
Ionic concentration c (mol/L)	1.31 × 10^{-3}	5.41 × 10^{-3}	4.03 × 10^{-4}	4.44 × 10^{-5}	1.75 × 10^{-5}	3.09 × 10^{-5}	4.15 × 10^{-5}
Ionic strength I (mol/L)	1.31 × 10^{-3}	5.41 × 10^{-3}	4.03 × 10^{-4}	4.44 × 10^{-5}	1.75 × 10^{-5}	3.09 × 10^{-5}	4.15 × 10^{-5}
Debye length κ^{-1} (nm)	12.9	4.9	15.1	31.4	50.4	42.2	30.5
Viscosity η (cp, 298 K)	1.65	3.31	0.89	0.803	0.945	1.996	1.65
$\Lambda^0_{salt}\eta$ (S cm^2 p mol^{-1})	0.825	0.96	1.068	0.683	0.576	0.659	0.545

[a)]. In NMF, the typical limiting molar ionic conductivity of a monovalent salt is between 41 to 65 S cm^2 mol^{-1} (41−51[13] S cm^2 mol^{-1}, 60−65[14] S cm^2 mol^{-1}, 35−49[15] S cm^2 mol^{-1}). Thus, we use Λ^0_{salt} = 50 S cm^2 mol^{-1} here as a reasonable estimation.
[b)]. In FA, the typical limiting molar ionic conductivity of a monovalent salt is between 26 to 31 S cm^2 mol^{-1} (26−31[16] S cm^2 mol^{-1}, 27−31[17] S cm^2 mol^{-1}). Thus, we use Λ^0_{salt} = 29 S cm^2 mol^{-1} here as a reasonable estimation.
[c)]. In DIW, we assume that the major ionic species are monovalent ions Li^+ (38.6 S cm^2 mol^{-1}), F^- (55.4 S cm^2 mol^{-1}), H^+ (349.8 S cm^2 mol^{-1}), and OH^- (198.6 S cm^2 mol^{-1}).[18] However, the contribution from H^+ and OH^- should be minor at pH neutral. Thus, we use Λ^0_{salt} = 120 S cm^2 mol^{-1} here as a reasonable estimation.
[d)]. In DMF, all the monovalent salts have very close limiting molar ionic conductivity, e.g. 83 [14] S cm^2 mol^{-1}, 79−93[19] S cm^2 mol^{-1}. Thus, we use Λ^0_{salt} = 85 S cm^2 mol^{-1} here as a reasonable estimation.
[e)]. In DMAc, the typical limiting molar ionic conductivity of a monovalent salt is between 44 to 77 S cm^2 mol^{-1}.[20] Thus, we use Λ^0_{salt} = 61 S cm^2 mol^{-1} here as a reasonable estimation.
[f)]. In DMSO, the typical limiting molar ionic conductivity of a monovalent salt is between 11 to 30 S cm^2 mol^{-1} (22−44 S cm^2 mol^{-1},[21] 20−45 S cm^2 mol^{-1}[22]). Thus, we use Λ^0_{salt} = 33 S cm^2 mol^{-1} here as a reasonable estimation.
g). In NMP, the typical limiting molar ionic conductivity of a monovalent salt is between 24 to 42 S cm^2 mol^{-1}.[23] Thus, we use Λ^0_{salt} = 33 S cm^2 mol^{-1} here as a reasonable estimation.

Table S2. Calculated Hamaker's constant (A) of $Ti_3C_2T_x$ in various solvents.

Solvent	ε_s	n_s	A (J)	A (k_BT)
NMF	171	1.43	4.26 × 10^{-20}	10.5
FA	109.5	1.45	4.13 × 10^{-20}	10.2
DIW	78.4	1.33	4.87 × 10^{-20}	12.0
DMF	37.2	1.43	4.16 × 10^{-20}	10.3
DMAc	37.8	1.44	4.10 × 10^{-20}	10.2
DMSO	46.7	1.48	3.88 × 10^{-20}	9.6
NMP	32.7	1.48	3.84 × 10^{-20}	9.5

Table S3. Comparison of THz Shielding performance between previous works and this work.

**Samples**	**Thickness (cm)**	**Density (g cm^{-3})**	**EMI SE (dB)**	**Frequency range (THz)**	**SSEt (dB cm^2 g^{-1})**	**Ref.**
NMF-film	0.000196	~3.00	54.2	0.3-1.6	92017	This work
NMF-film/CNT	0.000213	~2.79	60.8	0.3-1.6	102413	This work
NMF-film-2/CNT	0.00005	~3.03	35	0.3-1.6	230756	This work
DIW-film	0.000165	~3.63	46.5	0.3-1.6	77596	This work
DIW-film/CNT	0.000169	~3.06	48.3	0.3-1.6	93356	This work
DMF-film	0.000246	~2.12	44.6	0.3-1.6	85436	This work
DMF-film/CNT	0.000239	~2.32	50.2	0.3-1.6	90214	This work
Graphene/PMMA	0.0033	NA	60	0.2-2	300000	[24]
$Ti_3C_2T_x$ foam	0.0085	0.11	51	0.2-2	53500	[25]
$Ti_3C_2T_x$ film	0.001	2.8	46.7	0.2-2	14000	[25]
Graphene foam-1500	0.3	0.0007	50	0.1-1.6	238095	[26]
MWCNTs/graphene foam-1500	0.3	0.003	40	0.1-1.6	44444	[26]
Fe_3O_4/Graphene	0.2	NA	38	0.002-2.5	80000	[27]
Kapton-derived carbon	0.0125	~1.28	~70	0.2-0.5	~4360	[28]
CNF composite coatings	0.004-0.01	>1.17	32	0.57-0.63	677.5	[29]
OLC-PMMA nanocomposite	0.012	~1.18	4	0.1-3	~283	[30]
CNW-PMC composite	0.007	~1.10	40	0.57-0.63	5200	[31]
MWCNTs/PMMA	0.03	~1.18	20	0.1-4	~563	[32]
SWCNTs/PVA	0.03	~1.27	29	0.3-2.1	~523	[33]
MXene metamaterials	0.00005	/	~30	0.5-2	/	[34]
MXene waterborne paint	0.00383	/	50.5	0.2-1.6	/	[35]
MEB	0.0011	~2.92	50	0.2-1.2	~15556	[36]

References

[1] W. Li, Z. Zhang, B. Han, S. Hu, Y. Xie, G. Yang, *J. Phys. Chem. B* **2007**, *111*, 6452-6456.
[2] D. S. Gill, J. P. Singla, R. C. Paul, S. P. Narula, *J. Chem. Soc, Dalton Trans.* **1972**, 522-524.
[3] A. S. Dukhin, P. J. Goetz, *Characterization of liquids, dispersions, emulsions, and porous materials using ultrasound*, Elsevier, **2017**.
[4] R. J. Hunter, *Foundations of colloid science*, Oxford university press, **2001**.
[5] J. Lao, R. Lv, J. Gao, A. Wang, J. Wu, J. Luo, *ACS Nano* **2018**, *12*, 12464-12471.
[6] G. S. Park, D. H. Ho, B. Lyu, S. Jeon, D. Y. Ryu, D. W. Kim, N. Lee, S. Kim, Y. J. Song, S. B. Jo, *Sci. Adv.* **2022**, *8*, eabl5299.
[7] S. Wan, X. Li, Y. Wang, Y. Chen, X. Xie, R. Yang, A. P. Tomsia, L. Jiang, Q. Cheng, *science* **2021**, *374*.
[8] J. Ilavsky, P. R. Jemian, *J. Appl. Crystallogr.* **2009**, *42*, 347-353.
[9] G. Beaucage, *J. Appl. Crystallogr.* **1995**, *28*, 717-728.
[10] Y. Liu, M. Paskevicius, M. V. Sofianos, G. Parkinson, S. Wang, C.-Z. Li, *Fuel* **2021**, *292*, 120384.
[11] P. Pfeifer, F. Ehrburger-Dolle, T. P. Rieker, M. T. Gonzalez, W. P. Hoffman, M. Molina-Sabio, F. Rodriguez-Reinoso, P. W. Schmidt, D. J. Voss, *Phys Rev Lett* **2002**, *88*, 115502.
[12] H. D. Bale, P. W. Schmidt, *Phys. Rev. Lett.* **1984**, *53*, 596-599.
[13] I. Banik, M. N. Roy, *J. Chem. Eng. Data* **2013**, *58*, 3378-3386.
[14] S. Dožić, M. Vraneš, N. Zec, S. Gadžurić, *J. Mol. Liq.* **2014**, *195*, 99-104.
[15] R. Gopal, O. Bhatnagar, *J. Phys. Chem.* **1966**, *70*, 3007-3008.
[16] J. Thomas, D. F. Evans, *J.Phys. Chem.* **2002**, *74*, 3812-3819.
[17] P. C. Carman, *Journal of Solution Chemistry* **1978**, *7*, 845-858.
[18] K. Laidler, J. Meiser, Menlo Park CA, **1982**.
[19] P. B. Ohm, C. Asato, A. S. Wexler, C. S. Dutcher, *J. Phys. Chem. A* **2015**, *119*, 3244-3252.
[20] D. Das, B. Das, D. K. Hazra, *J. Solution Chem.* **2003**, *32*, 77-83.
[21] N. P. Yao, D. Bennion, *J. Electrochem.Soc.* **1971**, *118*, 1097.
[22] P. M. McDonagh, J. F. Reardon, *J. Solution Chem.* **1998**, *27*, 675-683.
[23] M. D. Dyke, P. G. Sears, A. I. Popov, *J. Phys. Chem.* **1967**, *71*, 4140-4142.
[24] C. Pavlou, M. G. Pastore Carbone, A. C. Manikas, G. Trakakis, C. Koral, G. Papari, A. Andreone, C. Galiotis, *Nat. Commun.* **2021**, *12*, 4655.
[25] Z. Lin, J. Liu, W. Peng, Y. Zhu, Y. Zhao, K. Jiang, M. Peng, Y. Tan, *ACS Nano* **2020**, *14*, 2109-2117.
[26] Z. Huang, H. Chen, S. Xu, L. Y. Chen, Y. Huang, Z. Ge, W. Ma, J. Liang, F. Fan, S. Chang, Y. Chen, *Adv. Opt. Mater.* **2018**, *6*, 1801165.
[27] H. Chen, Z. Huang, Y. Huang, Y. Zhang, Z. Ge, W. Ma, T. Zhang, M. Wu, S. Xu, F. Fan, S. Chang, Y. Chen, *ACS Appl. Mater. Interfaces* **2019**, *11*, 1274-1282.
[28] S. Venkatachalam, D. Bertin, G. Ducournau, J. F. Lampin, D. Hourlier, *Carbon* **2016**, *100*, 158-164.
[29] A. Das, C. M. Megaridis, L. Liu, T. Wang, A. Biswas, *Appl. Phys. Lett.* **2011**, *98*, 174101.
[30] J. Macutkevic, R. Adomavicius, A. Krotkus, D. Seliuta, G. Valusis, S. Maksimenko, P. Kuzhir, K. Batrakov, V. Kuznetsov, S. Moseenkov, O. Shenderova, A. V. Okotrub, R. Langlet, P. Lambin, *Diam. Relat. Mater.* **2008**, *17*, 1608-1612.
[31] A. Das, T. M. Schutzius, C. M. Megaridis, S. Subhechha, T. Wang, L. Liu, *Appl. Phys. Lett.* **2012**, *101*, 243108.

[32] J. Macutkevic, D. Seliuta, G. Valusis, R. Adomavicius, A. Krotkus, P. Kuzhir, A. Paddubskaya, S. Maksimenko, V. Kuznetsov, I. Mazov, I. Simonova, *Diam. Relat. Mater.* **2012**, *25*, 13-18.

[33] D. Polley, A. Barman, R. K. Mitra, *Opt. Lett.* **2014**, *39*, 1541-1544.

[34] G. Choi, F. Shahzad, Y.-M. Bahk, Y. M. Jhon, H. Park, M. Alhabeb, B. Anasori, D.-S. Kim, C. M. Koo, Y. Gogotsi, M. Seo, *Adv. Opt. Mater.* **2018**, *6*.

[35] H. Wan, N. Liu, J. Tang, Q. Wen, X. Xiao, *ACS Nano* **2021**, *15*, 13646-13652.

[36] N. Liu, Q. Li, H. Wan, L. Chang, H. Wang, J. Fang, T. Ding, Q. Wen, L. Zhou, X. Xiao, *Nat. Commun.* **2022**, *13*, 5551.
